# Shuxie-1 Decoction Alleviated CUMS -Induced Liver Injury via IL-6/JAK2/STAT3 Signaling

**DOI:** 10.3389/fphar.2022.848355

**Published:** 2022-04-06

**Authors:** Mengting Zhang, Wanhong Wu, Caoxin Huang, Teng Cai, Nengjiang Zhao, Suhuan Liu, Shuyu Yang

**Affiliations:** ^1^ Research Studio of Traditional Chinese Medicine, The First Affiliated Hospital of Xiamen University, School of Medicine, Xiamen University, Xiamen, China; ^2^ Shandong First Medical University and Shandong Academy of Medical Sciences, Jinan, China; ^3^ Xiamen Diabetes Institute, The First Affiliated Hospital of Xiamen University, School of Medicine, Xiamen University, Xiamen, China; ^4^ Research Center for Translational Medicine, The First Affiliated Hospital of Xiamen University, School of Medicine, Xiamen University, Xiamen, China

**Keywords:** CUMS, liver injury, traditional Chinese medicine, IL-6/JAK2/STAT3 signaling, compound preparation

## Abstract

**Introduction:** Chronic stress has been shown to cause liver damage in addition to psychological depression. Besides, drug-induced liver injury is frequently caused by antidepressants. Shuxie-1 decoction (SX-1) is a formula of traditional Chinese medicine commonly used in nourishing liver blood, and relieving depression. However, the underlying molecular mechanism remains unclear. Therefore, this study was designed to explore the effects and mechanisms of SX-1 in treating chronic stress-induced depression as well as liver injury.

**Methods:** Chronic unpredictable mild stress (CUMS) was applied to male Wistar rats for 4 weeks, with or without administration of SX-1 at low-dose and high-dose for 6 weeks, using Fluoxetine (Flu) as a positive control. Body weight was monitored once every 2 weeks. In the sixth week, the sugar preference test and open field test were carried out to evaluate the depression status. After that, the serum and liver tissues were collected. The quality control of SX-1 decoctions and drug-containing serum was controlled by UHPLC-QE-MS. The cell viability was measured by Cell Counting Kit-8 (CCK8). Enzyme-linked immunosorbent assay (Elisa), Western Blot and immunohistochemistrical staining was obtained to detect the protein levels in the plasma and the hepatic tissues, respectively.

**Results:** CUMS led to decreased 1) body weight, 2) the preference for sugar water, 3) the desire to explore in open field, and increased serum levels of corticosterone. All these factors were completely reversed by SX-1 treatment. Hematoxylin-eosin staining (HE) showed that SX-1 improved the hepatocyte vacuolization in CUMS treated rats, decreased the serum levels of alanine aminotransferase (ALT) and the deposition of type I collagen (Col I) in hepatocytes as well. CUMS increased the levels of hepatic Interleukin-6 (IL-6), and provoked the activation of Janus kinase 2 (JAK2) and signal transducer and activator of transcription 3 (STAT3), which was abrogated by SX-1 treatment. Cobalt chloride (CoCl_2_) increased the protein expression of IL-6 and p-STAT3 in AML12 cells. Besides, nuclear pyknosis was observed under electron microscope, which were recovered after rat SX serum.

**Conclusion:** SX-1 effectively ameliorated CUMS-induced depression-like behaviors as well as hepatic injuries, probably by the blockade of hepatic IL-6/JAK2/STAT3 signaling.

## Introduction

Chronic stress, resulted from negative emotions or repeated exposure to environmental stressors, can cause adverse consequences on the immune system, digestive system and nervous system by activating the hypothalamic-pituitary-adrenal (HPA) axis. Mounting evidences have suggested that stress can cause liver damages in addition to emotional alterations, in which oxidative stress and inflammation might be involved (Youssef et al., 2013; Demirdaş et al., 2016; Duda et al., 2016; Miller et al., 2019; Kortam et al., 2021). Chronic stress induces high expression of IL-6, TNFα and IL-1β in mouse hippocampus (Guo et al., 2019). Meanwhile, these inflammatory factors have been shown to activate the JAK2/STAT3 signaling, which further exacerbate the inflammatory response in the liver, resulting in hepatocyte necrosis and apoptosis and eventually leading to liver injury accompanied by dramatic increase of serum transaminase level (Li et al., 2020).

Studies have shown that IL-6 is an important inducer of acute phase response and infection defense in the liver. Under acute inflammation, IL-6 will induce liver synthesis of liver acute protein secreted by neutrophils, monocytes, and macrophages, which is advantageous for physical response to infectious injury and inflammation. (Baumann and Gauldie, 1994; Gabay and Kushner, 1999). However, in the case of chronic inflammation, the continuous activation of IL-6 signaling pathway is harmful to the liver and may eventually lead to the development of liver tumors (Schmidt-Arras and Rose-John, 2016). Il-6 binds to the membrane-bound IL-6 receptor -α, which induces the formation of an isomer complex consisting of two IL-6 molecules, IL-6 receptor -α and IL-6 receptor subunit -β. Formation of this complex leads to activation of the JAK/STAT3 signaling pathway, which leads to transcription of STAT3 target genes (Johnson et al., 2018). Blockade of the activation of JAK2/STAT3 is considered to play an important role in the repair of liver injury (Ilamathi et al., 2016). However, apart from the allopathic therapeutic agents, there are not many effective therapeutic options available for the injuries.

Traditional medicine defines the liver as an organ with multiple functions, including relieving pressure. Thus, soothing the liver can ameliorate the discomforts caused by stress. In clinical practice, a variety of Chinese herbal medicines have been widely used to treat stress-induced liver injury via nourishing and soothing the liver (Jia et al., 2017; Chen et al., 2020). SX-1 decoction is made from the composition of Suanzaoren Decoction and Xiaoyao San, which is showed effective in treating emotion-related diseases such as irritability, insomnia and dreaminess etc. (Yi et al., 2007; Chen et al., 2015; Liu et al., 2020). However, the mechanisms remain to be elucidated. Besides, the prescription of SX-1 is effective against liver injury. Many researches have demonstrated the anti-liver injury effect of Xiaoyao San and Suanzaoren Decoction (Zhu et al., 2007; Chen et al., 2015; Zhou et al., 2021). In this study, a rat model of CUMS was established to mimic the psychological and health condition under various unknown pressures (Antoniuk et al., 2019). The effects and mechanisms of SX-1 on the CUMS-induced emotional- and hepatic alterations were studied. Moreover, the mechanism and effect of SX-1 have also been verified in cells.

## Materials and Methods

### Drug Administration

The botanical drugs used to prepare the SX-1 decoctions were purchased from the First Affiliated Hospital of Xiamen University. The ingredients are listed in Table 1. All herbs were pooled together and boiled twice with pure water. The two decoctions were mixed together and concentrated to 1.383 g/ml. This decoction is a hospital preparation. The treatment dosage for rats was 6.25 times that for patients based on the body weight (Nair et al., 2018). For instance, a patient needs 155 g herbs per 70 Kg body weight every day. Then, a rat needs 1.383 g/100 g herbs, which is the low dose of SX-1 (SXL). The high dose (SXH) is twice the low dose. In the same way, the daily dose of fluoxetine required for an adult (20 mg/70 Kg) was converted into the dose of fluoxetine for rats (0.1786 mg/100 g). Fluoxetine is diluted with pure water.

**TABLE 1:** The herbal composition of Shuxie-1 Decoction (SX-1).

Chinses Name	Scientific Name	Family	Drug Name	Dosage(g)
Suan Zao Ren	Ziziphus jujuba Mill	Rhamnaceae	Ziziphi spinosae semen	20
Fu Ling	Poria cocos (Schw.) Wolf	Polyporaceae	Poria	10
Chuan Xiong	Conioselinum anthriscoides ‘Chuanxiong'	Apiaceae	Chuanxiong rhizoma	3
Bai zi ren	Platycladus orientalis (L.) Franco	Cupressaceae	Platycladi semen	20
Zhi huang qi	Astragalus mongholicus Bunge	Fabaceae	Astragali radix praeparata cum melle	10
Zhi Mu	Anemarrhena asphodeloides Bunge	Asparagaceae	Anemarrhenae rhizoma	10
Tai zi shen	Pseudostellaria heterophylla (Miq.) Pax	Caryophyllaceae	Pseudostellariae radix	10
Wu Wei Zi	Schisandra chinensis (Turcz.) Baill	Schisandraceae	Schisandare chinensis fructus	10
Huang lian	Coptis chinensis Franch	Ranunculaceae	Coptidis rhizoma	5
Long yan rou	Dimocarpus longan Lour	Sapindaceae	Longan arillus	10
Yuan zhi	Polygala tenuifolia Willd	Polygalaceae	Polygalae radix	10
Chai hu	Bupleurum chinense DC.	Apiaceae	Bupleuri radix	6
Bai shao	Paeonia lactiflora Pall	Paeoniaceae	Paeoniae radix alba	10
Dan shen	Salvia miltiorrhiza Bunge	Lamiaceae	Salviae miltiorrhizae radix et rhizoma	10
Mu xiang	Dolomiaea costus (Falc.) Kasana and A.K.Pandey	Asteraceae	Aucklandiae radix	5
Zhi zi	Gardenia jasminoides J.Ellis	Rubiaceae	Gardeniae fructus	6

Wistar rats (300–350 g, Shanghai SLAC Laboratory Animal Co., Ltd.) were gavaged with the prepared low-dose SX-1 (Rat SX serum, n = 20) or distilled water (Rat con serum, n = 20) for 7 days. Abdominal aortic blood was collected 1 h after intragastric administration on day 7. Before blood collection, rats were intraperitoneally injected with chloral hydrate at a dose of 0.3 ml/100 g. After anesthesia, the abdominal cavity of the rats was opened surgically to find the abdominal aorta. Disposable blood collection needle and negative pressure blood collection vessel were used to collect blood from the abdominal aorta. and the serum were extracted from blood with the centrifuge at 3,000 rpm for 15 min. The rat SX serum and rat con serum were mixed well respectively, then sterilized in a 55°C water bath for 30 min and stored at -20°. l-Glutathione Reduced was purchased from APExBIO.

### UHPLC-QE-MS Measure for Shuxie-1 Decoctions and Drug-Containing Serum

All herbs of SX-1 were crushed with a mixer mill for 60 s at 60 Hz. 100 mg of sample was added to 500 μl of extracted solution which dissolved in 80% methanol containing 10 μg/ml of internal standard. After 30 s vortex, the samples were homogenized at 45 Hz for 4 min and sonicated for 1 h in ice-water bath. After placing 1 h in -40°C, the samples were centrifuged at 12,000 rpm (RCF = 13,800 (×g), R = 8.6 cm) for 15 min at 4°C. Finally, the supernatant was obtained and put in a fresh 2 ml tube for LC-MS/MS analysis. 400 μl of plasma sample were added to 40 μl of hydrochloric acid (2 mol/L), then the mixture was vortexed for 1 min and followed by incubated for 15 min at 4°C. The vortex and incubat cycle were repeated for 4 times. Add 1.8 ml acetonitrile, then the mixture was vortexed for 5 min and the samples were centrifuged at 12,000 rpm (RCF = 13,800 (×g), R = 8.6 cm) for 5 min at 4°C. 1,600 μl of supernatant was transferred to a fresh tube and nitrogen dried. The dried samples were reconstituted in 150 μl of 80% methyl alcohol containing 10 μg/ml of internal standard by vortex for 5 min. The constitution was then centrifuged at 12,000 rpm (RCF = 13,800 (×g), R = 8.6 cm) for 5 min at 4°C, and 120 μl of supernatant was transferred to a fresh glass vial for LC/MS analysis.

**LC-MS/MS analysis of supernatant of SX-1** was performed on an Agilent ultra-high performance liquid chromatography 1290 UPLC system with a Waters UPLC BEH C18 column (1.7 μm 2.1*100 mm). The column temperature was set at 55°C and the sample injection volume was set at 5 μl. The flow rate was set at 0.5 ml/min. The mobile phase consisted of 0.1% formic acid in water (A) and 0.1% formic acid in acetonitrile (B). The multi-step linear elution gradient program was as follows: 0–11 min: 85–25% A; 11–12 min: 25–2% A; 12–14 min: 2–2% A; 14–14.1 min: 2–85% A; 14.1–15 min: 85–85% A; 15–16 min, 85–85% A. An Q Exactive Focus mass spectrometer coupled with an Xcalibur software was employed to obtain the MS and MS/MS data based on the IDA acquisition mode. During each acquisition cycle, the mass range was from 100 to 1,500, and the top three of every cycle were screened and the corresponding MS/MS data were further acquired. Sheath gas flow rate: 45 Arb, Aux gas flow rate: 15 Arb, Capillary temperature: 400°C, Full ms resolution: 70,000, MS/MS resolution: 17,500, Collision energy: 15/30/45 in NCE mode, Spray Voltage: 4.0 kV (positive) or -3.6 kV (negative).

**LC-MS/MS analysis of rat serum** was performed on an UHPLC system (Vanquish, Thermo Fisher Scientific) with a Waters UPLC BEH C18 column (1.7 μm 2.1*100 mm). The flow rate was set at 0.4 ml/min and the sample injection volume was set at 5 μl. The mobile phase consisted of 0.1% formic acid in water (A) and 0.1% formic acid in acetonitrile (B). The multi-step linear elution gradient program was as follows: 0–3.5 min: 95–85% A; 3.5–6 min: 85–70% A; 6–6.5 min: 70–70% A; 6.5–12 min: 70–30% A; 12–12.5 min: 30–30% A; 12.5–18 min: 30–0% A; 18–25 min: 0–0% A; 25–26 min: 0–95% A; 26–30 min: 95–95% A. An Orbitrap Exploris 120 mass spectrometer coupled with an Xcalibur software was employed to obtain the MS and MS/MS data based on the IDA acquisition mode. During each acquisition cycle, the mass range was from 100 to 1,500, and the top four of every cycle were screened and the corresponding MS/MS data were further acquired. Sheath gas flow rate: 30 Arb, Aux gas flow rate: 10 Arb, Ion Transfer Tube Temp: 350°C, Vaporizer Temp: 350°C, Full ms resolution: 60,000, MS/MS resolution: 15,000, Collision energy: 16/38/42 in NCE mode, Spray Voltage: 5.5 kV (positive) or -4 kV (negative).

### Animals and Treatments

Male Wistar rats (8 weeks old, 250–280 g) were purchased from Shanghai SLAC Laboratory Animal Co., Ltd. All the rats were housed under the controlled environment with a room temperature at 22 ± 2°C, a 12 h light/dark cycle, and with unlimited access to water and food. All animal experiments were carried out in accordance with the rules for the Laboratory Animal Ethics Committee of Xiamen University.

One week after the acclimation of the environment, the rats were randomly divided into the control group (n = 10) and CUMS model group (n = 40). For the control group, five rats were kept in each cage, and for the model group, each rat was housed in a single cage. CUMS was applied to the model group rats for 4 weeks. Different physical stimulation was applied to the CUMS model rat daily for 4 weeks, as summarized in Table 2. Four weeks later, the CUMS rats were further randomly divided into four groups: 1) CUMS rats treated with vehicle (CUMS), 2) CUMS rats treat with SX-1 at low dose (CUMS + SXL), 3) CUMS rats treat with SX-1 at high dose (CUMS + SXH), and 4) CUMS rats treat with Fluoxetine (CUMS + Flu). There are ten rats in each group (n = 10). SX-1 and Fluoxetine were administrated by gastric gavage with the volume of 0.1 ml/10 g for 6 weeks. Meanwhile, a small amount of stimulation was continued. After 6-week treatment, sucrose preference experiment and open field test were arranged to carry out. At the end of the experiments, the rats were anesthetized with 4% chloral hydrate. Blood and liver were collected under anesthesia. The lower right edge of the hepatic lobar was collected and fixed in 4% Paraformaldehyde (PFA), and the rest of the liver was cut into small pieces and frozen in a cryopreserved tube with liquid nitrogen, and then stored at -80 C.

**TABLE 2:** Daily stimulations summary from Day 1 to Day 28.

Day	Stimulations	Day	Stimulations	Day	Stimulations	Day	Stimulations
1	All night lighting	8	Restraint stress by restraint cages, 1 h	15	Clamps hold the tail, 2min✕2	22	Tilting the cage + Fasting and water
2	Restraint stress by restraint cages,30min	9	All night lighting	16	Restraint stress by restraint cages, 2 h	23	Restraint stress by restraint cages, 3 h
3	Damp bedding	10	Swimming in cold water, 4°C, 5min	17	Swimming in cold water, 4°C, 5min	24	Noise + All night lighting
4	Fasting and water	11	Fasting and water	18	Fasting and water	25	Fasting and water
5	Reversed day and night	12	All night lighting	19	heat stress, 3min	26	Damp bedding
6	Clamps hold the tail, 2min✕2	13	Damp bedding	20	Clamps hold the tail, 3min✕2	27	Swimming in cold water, 4°C, 5min
7	Damp bedding	14	Tilting the cage + No water	21	All night lighting	28	Restraint stress by restraint cages, 1 h

### Sucrose Preference Experiment

Sucrose preference experiment is a behavioral experiment to measure whether animals lack pleasure. When given the choice of drinking sucrose solution or water, rodents will choose to drink sucrose solution. Animals that don’t show this preference are thought to exhibit behavior similar to anhedonia (Papp et al., 1991). Sucrose preference experiment consists of two parts: adaptation training and test. During the training, two bottles of 1% (w/v) sucrose solution were put into each cage in the first 24 h, and then one of the bottles was replaced with pure water in the next 24 h. After adaptation, the rats were fasted for 24 h, and then the baseline test was started for 12 h. In the test, the rats could only choose two bottles weighed in advance, one is 1% (w/v) sucrose solution, and the other is pure water. After fasting for 12 h, the two bottles were taken away and weighed. The total liquid consumption, sugar consumption and pure water consumption were recorded. The formula of sugar preference index is as follows:

Sugar preference index %=sugar water consumption/(sugar water consumption+pure water consumption)×100%

### Open Field Test

One of the most commonly used behavioral tests for rodents is the open field test, which is one of the common methods to observe whether animals have the desire to explore (Sturman et al., 2018). The open field reaction box has a gray inner wall, with a digital camera mounted directly above it. The vision field of the camera covered the entire open field. The rats were allowed to adapt to the environment first. The central region and the peripheral region are set up in the small animal recording behavior analysis system I Topscanlite (Clever Sys). At the beginning of the experiment, the rats were gently placed in the center of the open field with their heads facing one of the four corners of the box. The timing was 5 min from the time when the rats entered the open field box. The system will record the residence time of the rats entering the central area and the total travel distance. At the end of each experiment, urine and stool were removed with 70% alcohol and dried with an air blower.

### AML12 Cell Culture

AML12 cells (Cellcook, Guangzhou, China) were cultured in DMEM/F-12 medium (BasalMedia, Shanghai, China) containing 10% fetal bovine serum (FBS, Gibco, Australia), 1% ITS liquid media supplement (Sigma, I3146), 1% Penicillin-Streptomycin (BasalMedia, Shanghai, China) and Dexamethasone 40 ng/ml in a 37°C 5% CO2 saturated humidity incubator. The normally cultured AML12 cell lines were centrifuged at 1,000 rpm for 5 min, and 10^5^ cells/well were placed in a 12-well plate and 5*10^3^ cells/well were placed in a 96-well plate and incubated at 37°C.

### Detection of AML12 Cell Viability by CCK8

AML12 cell culture was conducted in the following groups: a normal control group, rat con serum groups at [10%, 20%, 40%, 80%], rat SX serum groups at [10%, 20%, 40%, 80%], l-Glutathione (Glu) groups at [10 μM, 20 μM, 40 μM, 80 μM], stattic groups at [5 μM, 10 μM, 20 μM], cobalt chloride (CoCl_2_) groups at [200 μM, 400 μM, 800 μM]. Glu used in this study was provided by APExBIO (Houston, USA; CAS No. 70-18-8; Molecular formula: C_10_H_17_N_3_O_6_S; Molecular weight: 307.32); l-Glutathione Reduced is an endogenous antioxidant, with the ability to scavenge oxygen-derived free radicals, and stattic was provided by Selleck (Shanghai, China; CAS No. 19983-44-9; Molecular formula: C_8_H_5_NO_4_S; Molecular weight: 211.19), and CoCl_2_ was provided by Sigma (Darmstadt, Germany; CAS No. 7646-79-9; A string у Split spinning: CoCl_2_; Molecular weight: 129.84). After 80% cell adhesion, different concentrations of rat con serum and rat SX serum were added. The system of each pore was 100 µl. The experiment was repeated three times independently. After different time points of drug action, 10 μl cell counting kit-8 (CCK8) detection solution (MedChemExpress, Shanghai, China) was added to each pore, and the reaction time was 2.5 h at 37°C. A blank control group was set up and treated in the same way without anything added. Optical density (OD) was measured at 450 nm. The results were calculated using the following equation: Cell survival rate (%) = OD value of experimental group/OD value of non-drug group × 100%.

### Biochemical Assays and ELISA

Serum levels of ALT and alanine aminotransferase (AST) were measured by ALT and AST assay kit (IFCC) (140120008, 140220006, Shenzhen Mindray Bio-Medical Electronics Co., Ltd.) using an automatic biochemical detector (BS-240VET, Shenzhen Mindray Bio-Medical Electronics Co., Ltd.).

Serum levels of corticosterone were measured by commercial Elisa kits according to the manufacturer’s protocols (201074, R&D, U.S).

Hepatic IL-6 level was measured by ELISA. Briefly, the rat livers were homogenized with RIPA buffer and centrifuged at 10,000 rpm for 15 min at 4°C. The supernatant was collected and used for determination of the protein levels of IL-6, using commercial ELISA kits (ELR-IL-6-1, Ray Biotech, U.S) following the manufacturer’s protocols.

### Real Time Quantitative PCR Analysis

Total RNA was extracted from liver tissue using total RNA Extraction Kit (Tiangen, No. DP419). CDNA was synthesized from total RNA (2 μg) using fastking RT Kit (with gDNase) reverse transcription Kit (Tiangen, No. KR116-02). Real time quantitative PCR (qPCR) was performed using SYBR Green PCR Master Mix (Tiangen, No. FP205-02) and the forward primer of IL-1β was CAC​CTC​TCA​AGC​AGA​GCA​CAG, the reverse primer of IL-1β was GGG​TTC​CAT​GGT​GAA​GTC​AAC; the forward primer of IL-18 was TGG​AGA​CTT​GGA​ATC​AGA​CC, the reverse primer of IL-18 was GGC​AAG​CTA​GAA​AGT​GTC​CT; the forward primer of TNFα was AAA​TGG​GCT​CCC​TCT​CAT​CAG​TTC, the reverse primer of TNFα was TCT​GCT​TGG​TGG​TTT​GCT​ACG​AC, the forward primer of β-actin was CTG​TGT​GGA​TTG​GTG​GCT​CT, the reverse primer of β-actin was CAG​CTC​AGT​AAC​AGT​CCG​CC. Gene expression data were analyzed by fluorescence quantitative PCR (Roche Lightcycle 480II, USA).

### Western Blot Analysis

The rat livers and AML12 cells were lysed by RIPA buffer, separated by 10% polyacrylamide gel electrophoresis and then transferred to polyvinylidene fluoride (PVDF) membrane. After blocking in 5% Bovine Serum Albumin (BSA), the membrane was incubated with the phospho-JAK2 (Tyr931) (1:1,000, affinity), total JAK2 (1:1,000, proteintech), phospho-STAT3 (Tyr705) (1:1,500, abcam), total STAT3 (1:2000, abcam), IL-6 (1:500, bioworld), IL-18 (1:2000, bioworld), IL-1β (1:500, sino biological), TNFα (1:1,000, sino biological), HIF-1a (1:1,000, Novusbio), HIF-2a (1:1,000, Cell Signaling Technology), VEGFa (1:1,000, Novusbio) primary antibody seperately overnight at 4°C, and then was incubated with HRP conjugated secondary antibody for 1 h at room temperature. Western blots were displayed by advanced enhanced chemiluminescence kit and then semi-quantified by Clinx (Shanghai Qinxiang Scientific Instrument Co., Ltd).

### Histopathology and Immunofluorescence Staining

The liver tissue was fixed in tissue fixative, embedded in paraffin, and then sectioned (5 μm) for HE and Immunohistochemistrical staining. HE staining was used to observe the morphological changes of the liver. Hepatic fibrosis was visualized by Immunofluorescence staining of Col I. The monoclonal antibody against Col I (1:500, Servicebio) and secondary goat anti-rabbit IgG-HRP antibody (1:300, Servicebio were used respectively, and the positive signals were observed under the florescence microscope.

### Statistical Analysis

All data are shown as the mean ± standard error (SEM). Two tailed unpaired *t*-test was used to compare the two groups of data. Multiple groups of random measurement data were tested by one-way ANOVA. After conforming to the normality test, the data were continued to be used to test homogeneity of variance between groups, using least-significant difference LSD for multiple comparisons on the assumption of equal variance. Repeated measures ANOVA was applied to the data detected at multiple time points such as weight and depression-like behaviors. SPSS 25 software (IBM, United States) was used to analyze the data. The difference was considered statistically significant when *p* < 0.05.

## Results

### Quality Control of Shuxie-1 Decoctions and Drug-Containing Serum by UHPLC-QE-MS

**FIGURE 1:**
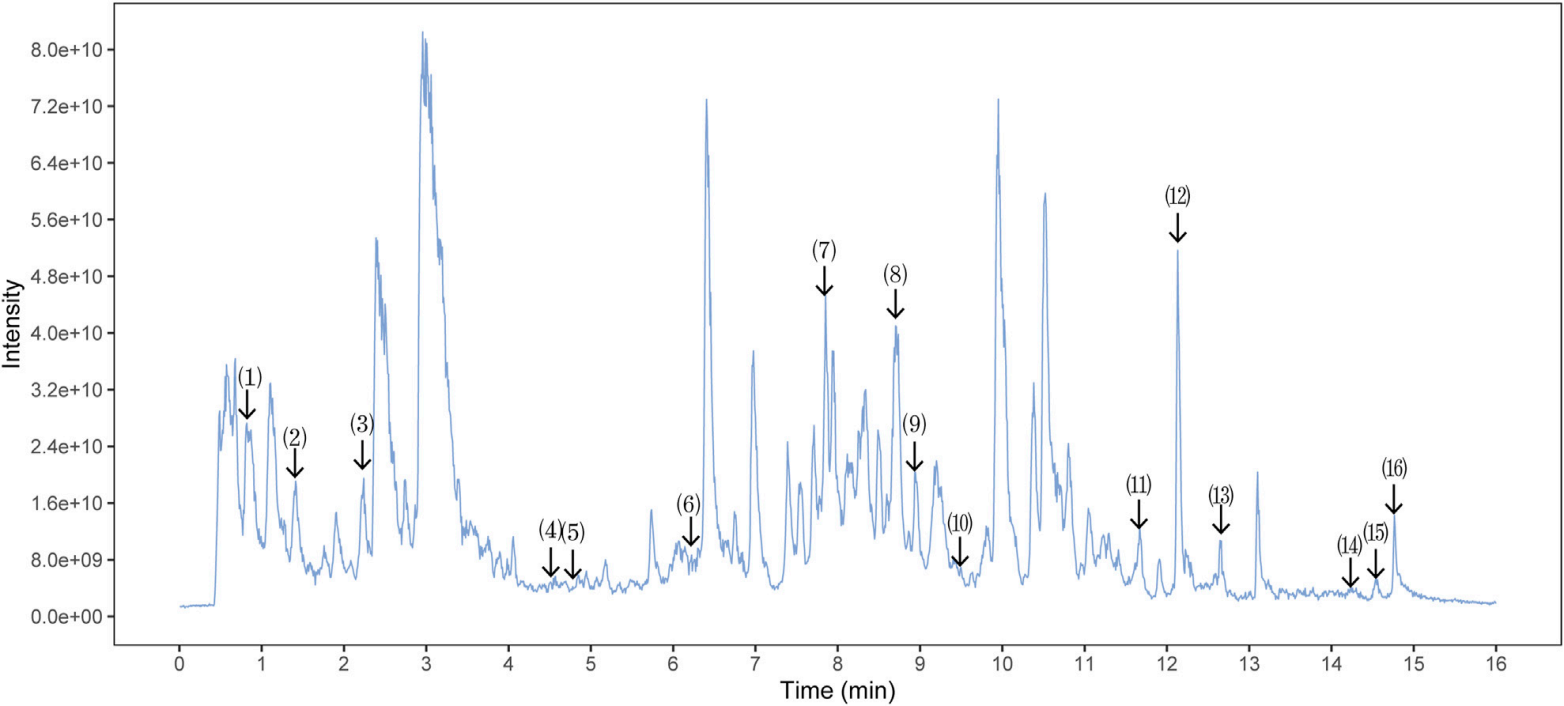
Ultra-High Performance Liquid Chromatography (UPLC) analysis of ingredients from the SX-1 sample (n = =3). The components on the chromatogram are as follows from left to right: (1) Mangiferin; (2) Paeoniflorin; (3) Epiberberine; (4) Heterophyllin B; (5) Polygalic acid; (6) Saikosaponin D; (7) Dehydrocostus lactone; (8) Tanshinone IIA; (9) Timosaponin A-III; (10) Poricoic acid A; (11) Ursolic acid; (2) Schisantherin A; (13) Jatrorrhizine; (14) Palmitic Acid; (15) Tetramethylpyrazine; (16) Betulinic Acid.

**FIGURE 2:**
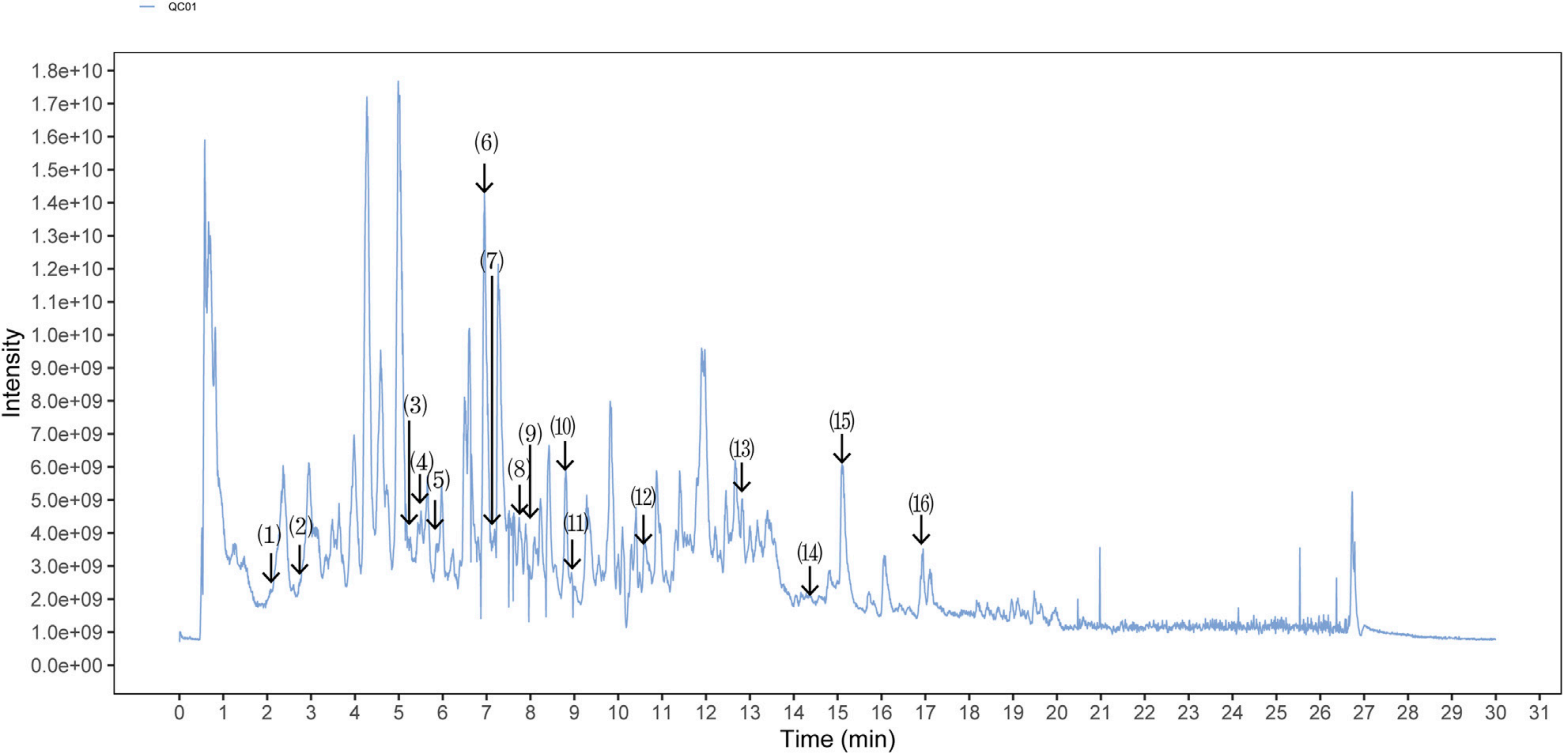
Ultra-High Performance Liquid Chromatography (UPLC) analysis of ingredients from the drug-containing serum (n = =3). Comparison strategy: rat con serum VS. rat SX serum. The components on the chromatogram are as follows from left to right: (1) Shanzhiside; (2) Ferulic Acid; (3) Paeoniflorin; (4) Picroside II; (5) Phellodendrine chloride; (6) Salvianolic acid A; (7) Tenuifoliside A; (8) Formononetine; (9) Podocarpusflavone A; (10) Timosaponin B II; (11) Heterophyllin B; (12) 5-[6-(3-hydroxy-4-methoxyphenyl)-1,3,3a,4,6,6a-hexahydrofuro [3,4-c]furan-3-yl]-2-methoxyphenol; (13) Poricoic acid A; (14) 2-[4,5-Dihydroxy-6-[[8-hydroxy-8a-(hydroxymethyl)-4,4,6a,6b,11,11,14b-heptamethyl-1,2,3,4a,5,6,7,8,9,10,12,14a-dodecahydropicen-3-yl]oxy]-2-[[3,4,5-trihydroxy-6-(hydroxymethyl)oxan-2-yl]oxymethyl]oxan-3-yl]oxy-6-methyloxane-3,4,5-triol; (15) Betulinic Acid; (16) Palmitic Acid.

The quality control of SX-1 and drug-containing serum were investigated by UHPLC-QE-MS. The UHPLC-QE-MS chromatogram is shown in Figure 1 and Figure 2. Several ingredients were identified. As shown in Figure 1 and Figure 2, sixteen compounds were distinguished respectively.

### SX-1 ameliorated CUMS-Induced Behavior Alterations and Decreased Serum Level of Stress Hormone Corticosterone

**FIGURE 3:**
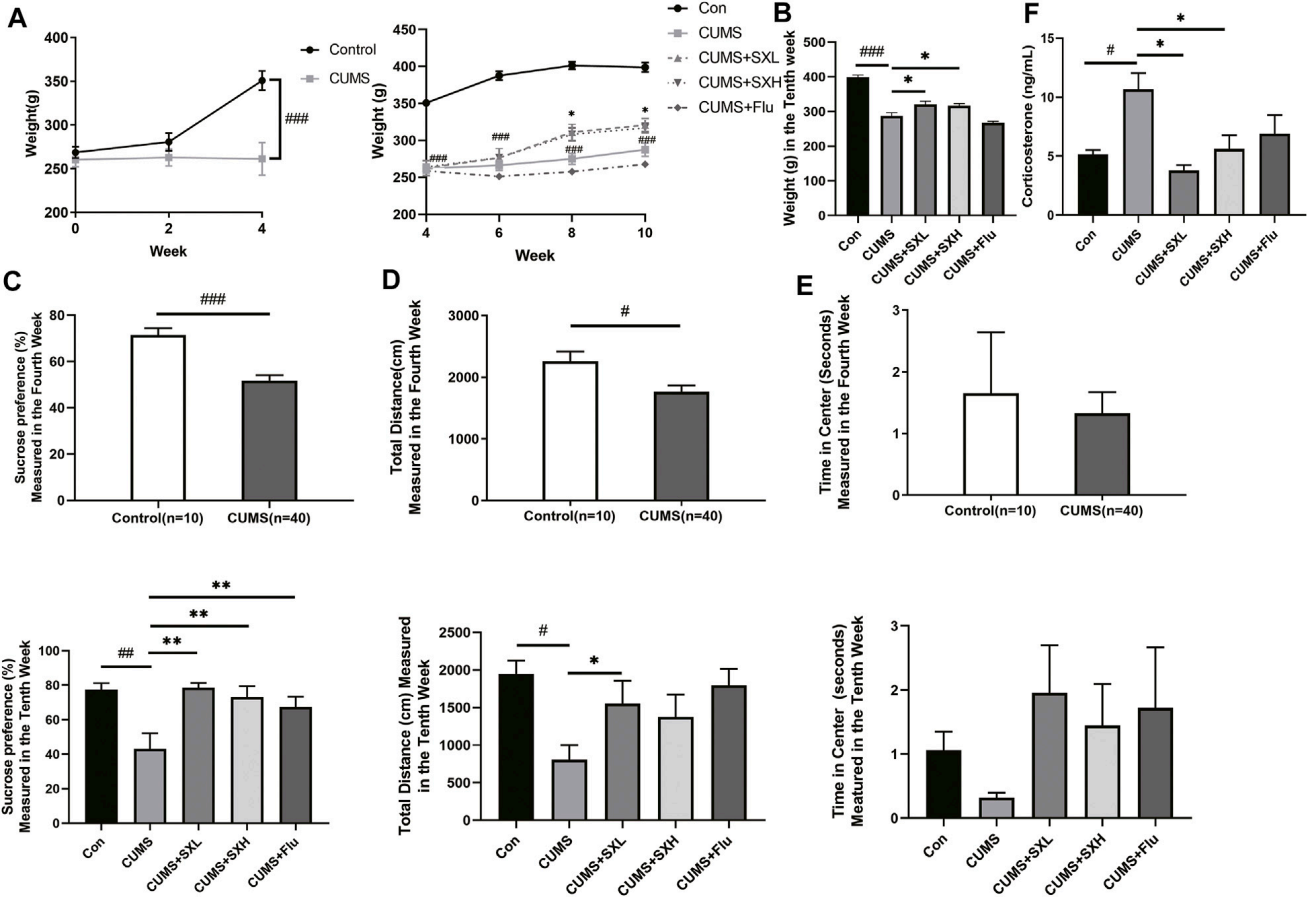
Effects of SX-1 on body weight, behaviors and serum corticosterone (n = 7–40). The body weight of rats was measured once every 2 weeks **(A)**. After 6 weeks of SX-1 treatment, the body weight **(B)**, sucrose preference test **(C)** and open field test (OPT) were performed. The total moving distance **(D)** and exploration time in center **(E)** within 5 min of each rat were recorded. Rat serum was used to determine the level of corticosterone **(F)**. #: compared to control group (Con); *: compared to CUMS group.

Four-week CUMS significantly reduced the rat body weight (*p*<0.001), which was maintained through the following 6-weeks treatment. SX-1 significantly restored the CUMS-resulted decreased body weight at both low and high doses (*p =* 0.004 and *p* = 0.032, respectively), while Flu had no effect (*p =* 0.259; Figures 3A,B). CUMS provoked obvious depression-like behaviors, indicated by monitoring of sucrose preference and total activity distance in open field test (both *p*<0.001), which were improved by SX-1 and Flu (*p*<0.001 and *p* = 0.01; Figure 3C) and SXL respectively (*p* = 0.021; Figure 3D). SX-1 also tended to recover the exploration time length in central area, although without statistical significance (*p* = 0.928 or *p* = 0.812; Figure 3E). Consistently, CUMS significantly increased the serum levels of stress-related hormone corticosterone compared with the control group, which were completely reversed by SX-1 but not Flu (*p* = 0.001, *p*<0.001, *p* = 0.003 and *p* = 0.051, respectively; Figure 3F).

### SX-1 ameliorated CUMS-Induced Hepatic Injuries

**FIGURE 4:**
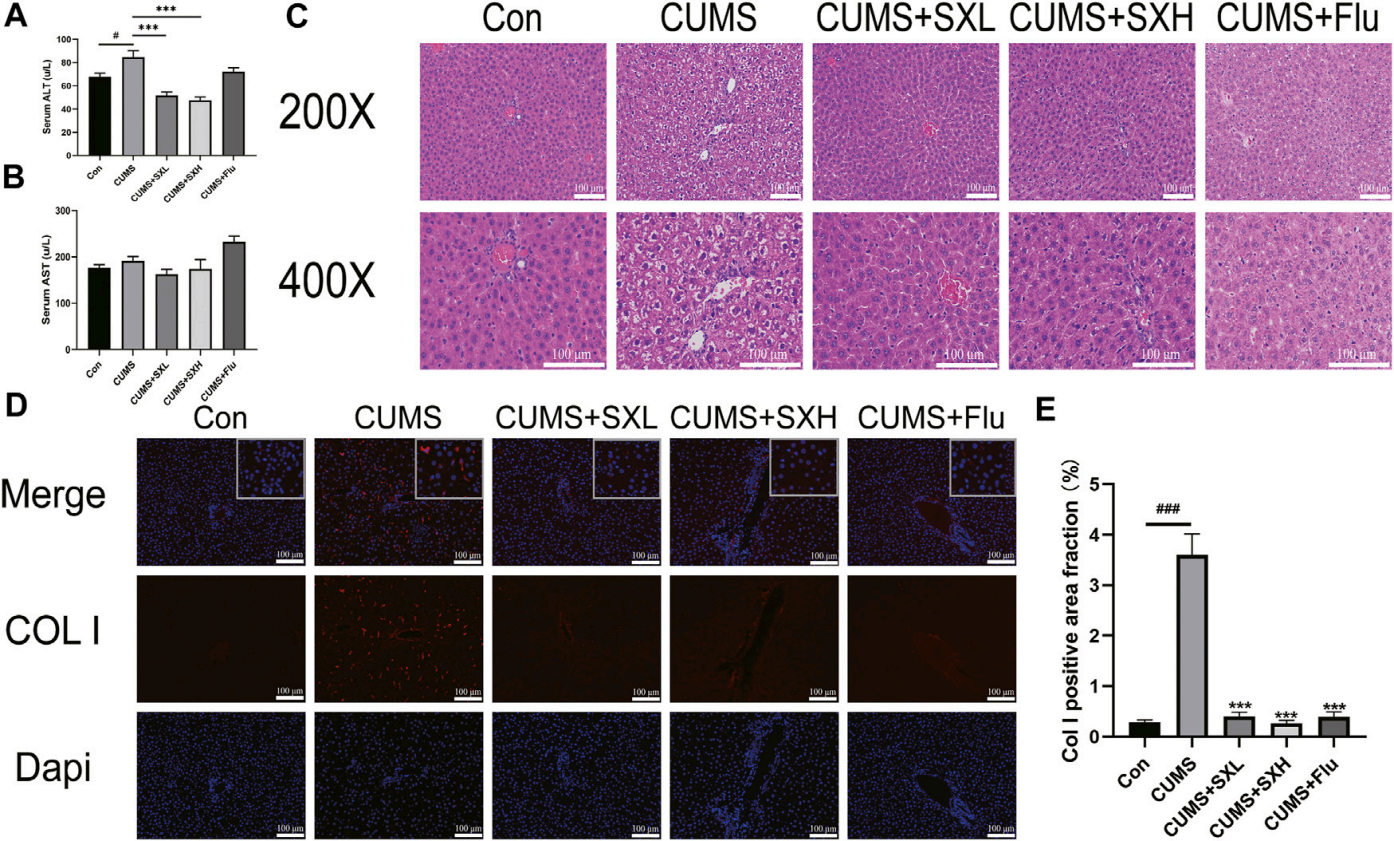
Histopathology and immunofluorescence for Col I in the hepatic tissues (n = 3–9). After 6 weeks of SX-1 treatment, the serum of rats was taken to determine ALT and AST **(B–C)**. Then, the right lower edge of the liver lobe of each rat was fixed, it was embedded in paraffin and sectioned (5 μm). Then the sections were stained with H&E **(A)** and immunofluorescence for Col I **(D)**. The positive area fraction of Col I was shown in **(E)**. ###: compared to control group (Con); ***: compared to CUMS group.

As an indicator of hepatic injury, serum levels of ALT were significantly increased in CUMS rats compared to that of the control rats (*p* = 0.003), which were completely reversed by SX-1 but not Flu (*p* < 0.001 and *p* = 0.063; Figure 4A). The serum levels of AST were of no difference among groups (*p* = 0.142, *p* = 0.184, *p* = 0.409 and *p* = 0.073, respectively; Figure 4B). HE staining showed that CUMS resulted in obviously disordered hepatic sinus and lobule structure, with typical balloon-like changes, which were improved by SX-1 treatment (Figure 4C).

### SX-1 Reversed CUMS-Induced Hepatic Fibrosis

CUMS induced a significant deposition of Col I in the liver. As shown in Figures 4A,D large number of red fluorescence surrounded the nucleus in the CUMS group, that is, the pathological manifestation of Col I deposition in the extracellular matrix. It also indicated the development of hepatic fibrosis. After SX-1 and Flu treatment, the red fluorescence in the picture decreased significantly. According to the statistics of fluorescence area by ImageJ software, SX-1 and Flu can significantly reverse the pathological manifestations of liver fibrosis (*p* < 0.001) (Figures 4D,E).

### SX-1 Inhibited CUMS-Activated IL-6/JAK2/STAT3 Signaling

**FIGURE 5:**
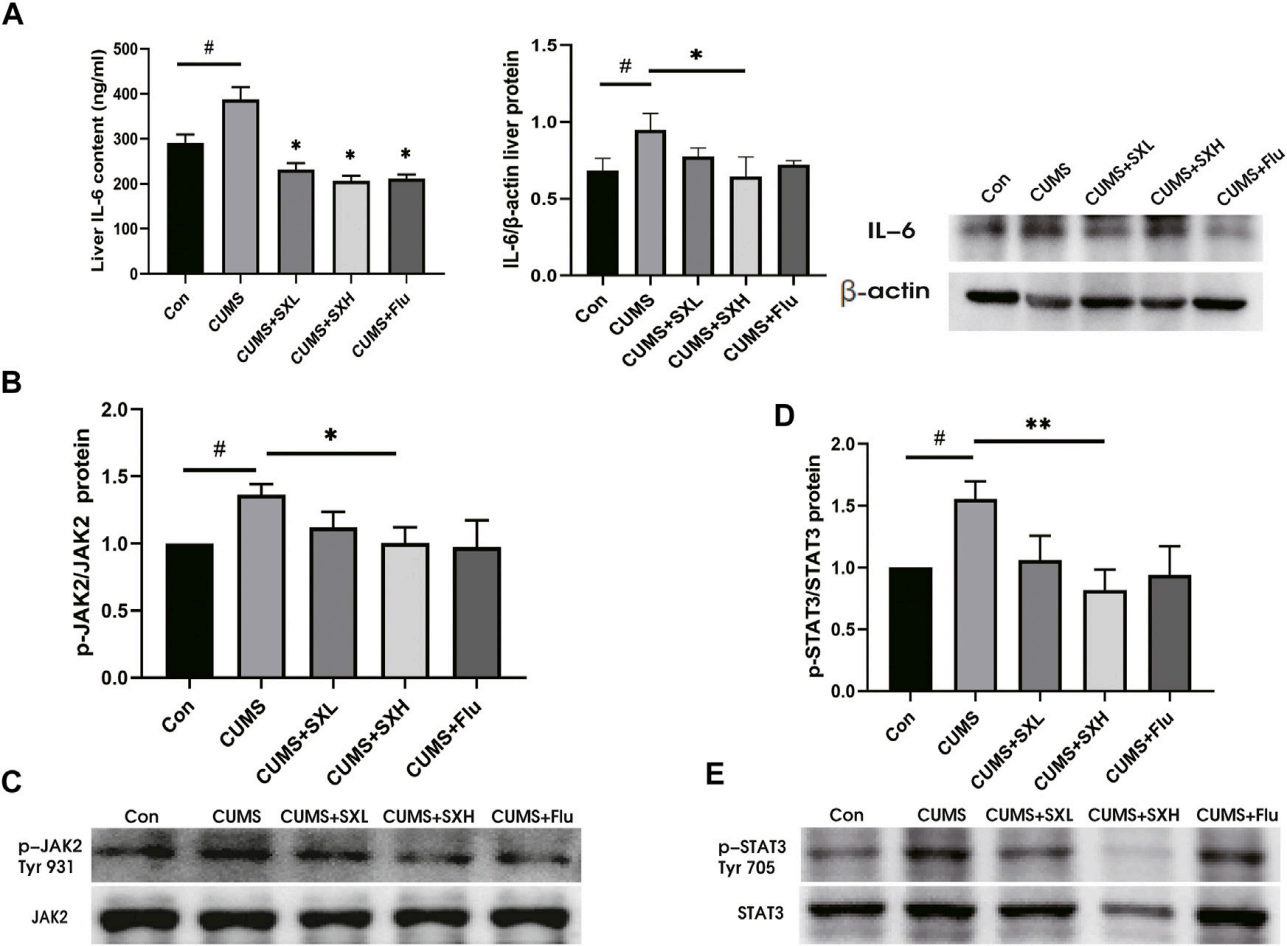
IL-6 protein content and JAK2 / STAT3 phosphorylation in liver tissue (n = =5–-7). IL-6 in liver tissue was detected by ELISA kit and western blot **(A)**. p-JAK2 (Tyr 931) / p-STAT3 (Tyr 705) and total JAK2 / STAT3 were determined by Western blot. The ratio of p-JAK2 / JAK2 **(B–-C)** and p-STAT3 / STAT3 **(D–-E)** reflected the phosphorylation level of the protein. #: compared to control group (Con); *: compared to CUMS group.

At both mRNA and protein levels, CUMS up-regulated the inflammatory factors such as IL-1β, IL-18 and TNFα in liver tissues, which were tended to be restored by SX-1, although without statistical significance (both *p*>0.05; Supplementary Figure S1). Critically, compared with the control rat, CUMS rats showed increased levels of hepatic IL-6 protein, which were reversed by SX-1 and Flu treatment in Elisa test (both *p*<0.05; Figure 5A left). However, in WB, the protein level of IL-6 decreased significantly in SXH compared with CUMS (*p* = 0.027; Figure 5A right). Accordingly, the phosphorylation of JAK2 and STAT3 were significantly up-regulated by CUMS (*p* = 0.044 and *p* = 0.027, respectively), indicating the activation of IL-6/JAK2/STAT3 signaling, which were significantly improved by SXH treatment (*p* = 0.046 and *p* = 0.005; Figures 5B–D).

### Mechanism Verification of SX-1 Alleviating Hepatocyte Injury in AML12 Cells

*In vivo*, we also observed that CUMS induced the expression of HIF-2α in the liver rather than HIF-1α (Supplementary Figure S2A, B). HIF-2α was only expressed in specific cells, such as hepatocytes. At the same time, acute hypoxia rapidly induced HIF-1α and HIF-2α proteins accumulate in cells. However, with the prolongation of hypoxia time, the stability of HIF-1α mRNA decreased and its protein expression decreased, while HIF-2α protein expression continued to increase (Bartoszewski et al., 2019). Therefore, we tried to explore the mechanism of hypoxia, but VEGFa protein could not be activated (Supplementary Figure S2C), and the mechanism was not clear. Therefore, in order to further verify whether SX-1 could improve CUMS induced liver injury through IL-6/JAK2/STAT3 signaling pathway, we used CoCl_2_ induced cell hypoxia model *in vitro*, that cellular hypoxia increases the expression of inflammatory factors (Tang and Ran, 2018; Gu et al., 2021). In particular, HIF-2α increases the protein expression of IL-6, and it is HIF-2a rather than HIF-1a that further causes liver lipid accumulation (Qu et al., 2011).

**FIGURE 6:**
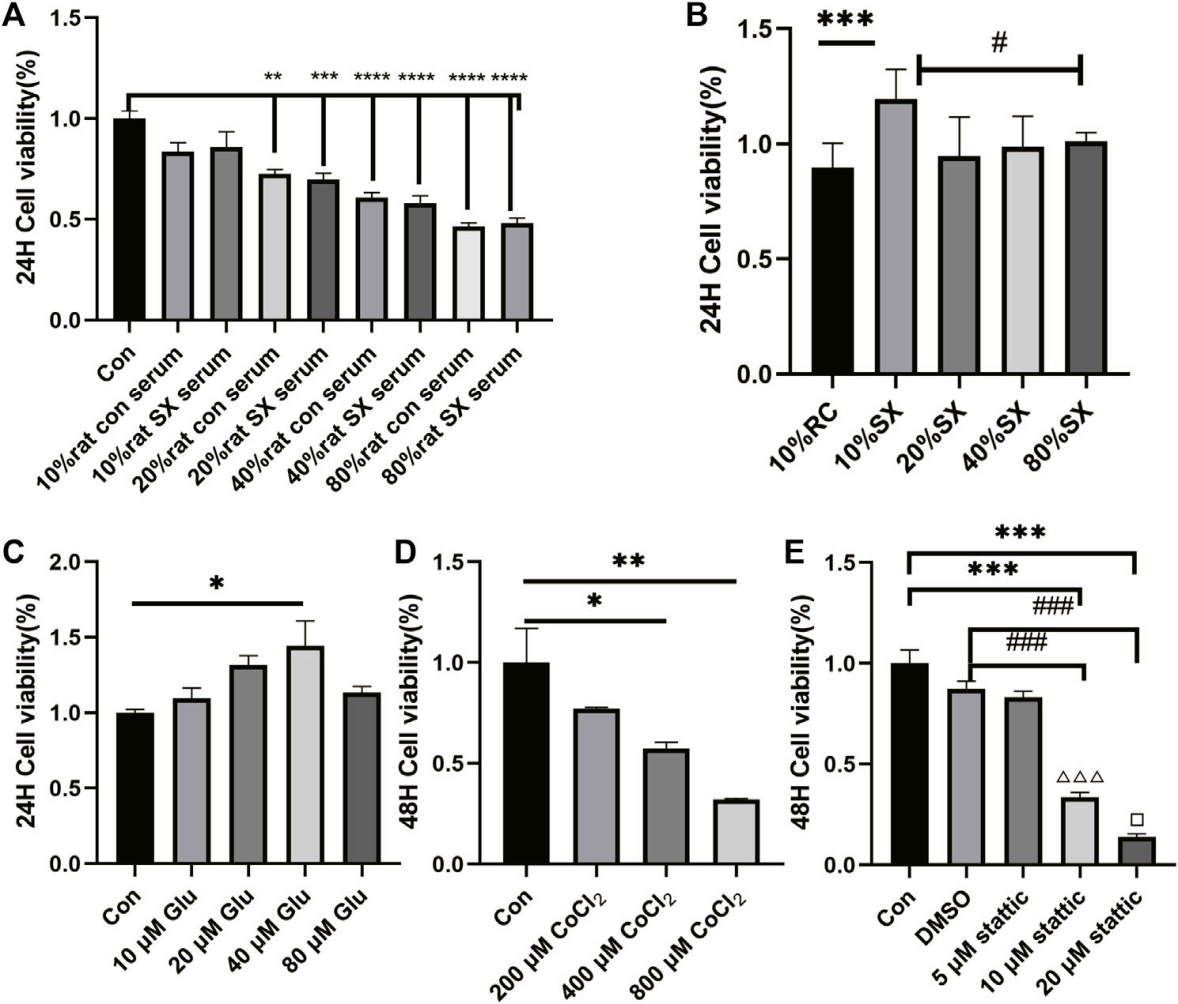
Effect of drug concentration on cell viability (n = 3). Cell viability was detected by CCK8 reagent. Drug concentration screening was carried out in the form of concentration gradient, including rat con serum **(A)**, SX drug containing serum **(B)**, Glu **(C)**, CoCl_2_ **(D)**, stattic **(E)**. *: compared to control group (Con);#: compared to 10% SX group; ###: compared to DMSO group; △△△: compared to 5 μM stattic; □: compared to 10 μM stattic.

Firstly, we applied CCK8 method to explore the cell concentration of drugs used in this validation experiment, such as SX-1, CoCl_2_, stattic and l-Glutathione (Glu). Compared with the control group, 10% rat serum had no effect on cell viability (*p* = 0.126; Figure 6A). Therefore, 10% rat con serum was used as the control in the subsequent groping for a suitable concentration of SX-1. Compared with 10% rat con serum, 10% SX-1 acting on cells for 24 h showed a protective effect (*p*<0.001; Figure 6B). Similarly, 40 μM Glu had a significant protective effect on cells compared with the control group for 24 h (*p* = 0.028; Figure 6C). While CoCl_2_ inhibited cell growth in a concentration dependent manner. When the concentration of CoCl_2_ was 400 μM, the cell viability was significantly lower than that of the control group for 48 h (*p* = 0.020; Figure 6D); while 10 μM stattic began to reduce cell viability when it was applied to cells for 48 h (*p*<0.001; Figure 6E).

**FIGURE 7:**
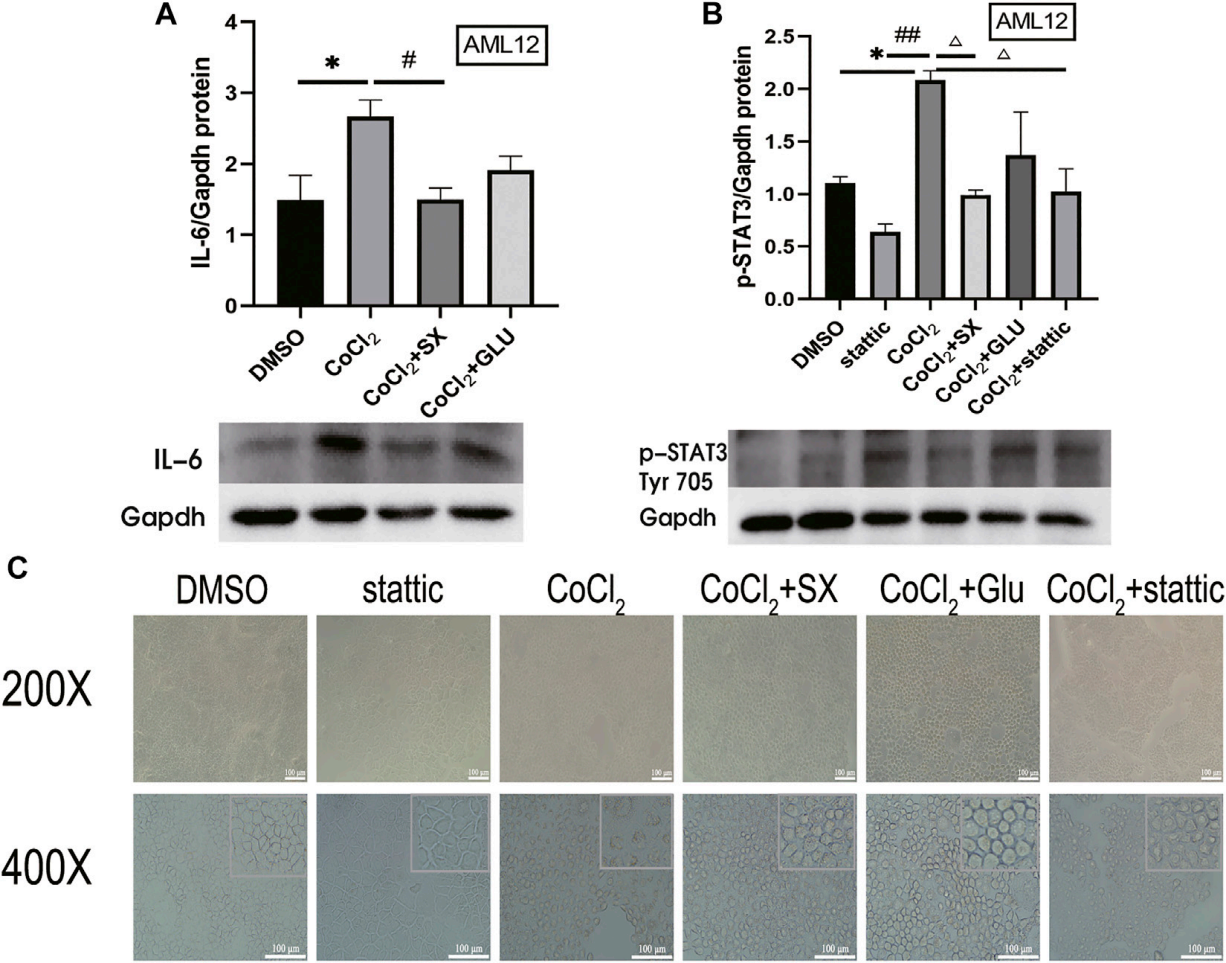
Protein expression of IL-6 and p-STAT3 in AML12 cells (n = 3). IL-6 **(A)** and p-STAT3 (Tyr 705) **(B)** in liver tissue was detected by western blot. The cell morphology was obtained under electron microscope **(C)**. *: compared to DMSO group; #: compared to CoCl_2_ group; ##: compared to stattic group; △: compared to CoCl_2_ group in figure B.

Then, we established AML12 cells hypoxia model with CoCl_2_, which increased HIF-2α and IL-6 level (*p* = 0.045 and *p* = 0.046, respectively), and then treated the cells with SX-1 drug-containing serum. SX-1 can significantly reduce HIF-2α and IL-6 level (*p* = 0.017, Supplementary Figure S3; *p* = 0.049, Figure 7A). Next, we used Stattic, a small molecule inhibitor that effectively inhibits STAT3 activation and nuclear translocation, to explore whether SX-1 has the effect of reducing STAT3 activation. Under the action of CoCl_2_, the level of p-STAT3 was significantly upregulated compared with the DMSO group (*p* = 0.004). However, after SX-1 and stattic intervention, both of them significantly reduced the expression of p-STAT3 (*p* = 0.002 and *p* = 0.003; Figure 7B). In accordance, we found that CoCl_2_ induced nuclear pyknosis and cell swelling in AML12 cells compared with the control group under electron microscope, while SX-1 and stattic intervention alleviated nuclear pyknosis and cell swelling to some extent (Figure 7C). This suggests that p-STAT3 is a key target of SX-1, which plays a role in saving hepatocyte injury by reducing the expression of p-STAT3.

## Discussions

In the theory of Traditional Chinese Medicine (TCM), liver plays a critical role in emotion regulation. Thus, physicians commonly treat many emotional disorders using liver as a therapeutic target. Here, we applied a TCM formula SX-1 decoction, which relieves depression and meanwhile soothes liver, to a rat model of chronic unpredictable mild stress (CUMS)-induced depression. The goal is to investigate the role of SX-1 in alleviating CUMS-induced liver damage and depression-like behavior and its mechanism. We found that CUMS did result in significant hepatic injuries, showed by increased serum levels of ALT and destructed hepatic structure, in addition to depression-like behaviors, showed by weakened sucrose preference- and open field test. SX-1 treatment significantly reversed CUMS-induced hepatic injuries as well as depression-like behaviors.

CUMS exposed the rats to a long-term and unfixed irritating condition, thus resulted in depression-like symptoms such as negative emotion, loss of interest, and weight loss. CUMS provided a stable model to investigate the effects and the mechanism of SX-1 on hepatic- and emotional impairments in depressed rats. As expected, CUMS led to increased serum levels of corticosterone, a stress indicator, suggesting that CUMS did exposed the rat in a stressed situation. We found that CUMS decreased body weight, reduced the rats’ interests in sugar water in the sucrose preference test, and reduced the autonomic activity and the desire to explore the central area in the open field test, showing a depression-like behaviour (Figure 3). Corticosterone is a stress hormone produced by HPA axis presenting hyperactivity under stress conditions. Depression is a typical example of chronic HPA axis hyperactivity (Chrousos and George, 2009), which is commonly recognized as a mental disorder. In TCM theory, liver is in charge of emotional activities. Thus chronic- or intensive emotional stimulus may damage the liver function, vice versa, hepatic dysfunction may also result in emotional alterations. It was reported that corticosterone could aggravate inflammation in the liver (Gao et al., 2019). In the present study, we did observe destructed hepatic structures, showed as disordered sinus and lobule structure and typical balloon-like changes, in CUMS treated rats. Serum levels of ALT, an indicator of hepatocyte damage, increased significantly in CUMS rats (Figures 4A–C). In addition, immunofluorescence staining showed increased expression of COL I protein in the liver of CUMS-treated rat, suggesting the deposition of collagen matrix in the liver and existence of fibrosis (Figures 4D,E). SX-1 significantly ameliorated all the above described emotional- and hepatic abnormalities induced by CUMS, while the positive control Flu only tended to have protective actions while did not reach any significance, indicating that the liver-targeting treatment by SX-1 is more effective than the serotonergic agent-Flu in treating CUMS-induced emotional and hepatic injuries.

SX-1 may alleviate liver injury by down-regulating IL-6 and JAK2/STAT3 pathway (Figure 5). IL-6 is a pleiotropic cytokine (Schmidt-Arras and Rose-John, 2016), and also a suitable stress biomarker (Quinn et al., 2018). On one hand, IL-6 is essential for continuous response of cortisol to chronic stress by activating JAK/STAT3 signaling cascade, thus mediating HPA axis plasticity, especially under chronic stress (Papanicolaou et al., 1996). On the other hand, hepatic IL-6 levels could be induced by epinephrine (van Gool et al., 1990). Therefore, IL-6 plays a key role in the development of stress susceptibility and related behaviors. In the present study, we found that CUMS upregulated the protein levels of IL-6 in the liver (Figure 4A). IL6 is extensively involved in liver injury. The IL6-treated rat liver showed increased collagen synthesis and severe liver cell necrosis, implying the involvement of IL-6 in the development of hepatitis and liver fibrosis (Choi et al., 1994). Here in our study, we did observe COL I deposition in hepatocytes. COL I is type I collagen, usually distributed in skin, tendon and other places, and belongs to fibroblast collagen. When COL I is present in the extracellular stroma, it indicates the deposition of extracellular matrix and the development of fibrosis. It is commonly accepted that IL-6 activated JAK2/STAT3 signaling plays a key role in the regulation of cell growth, differentiation, proliferation and immune function (Gu et al., 2011; Dang et al., 2019). Xiang and others reported that IL-6 can be regulated by Hepatic leukemia factor (HLF) transcription and enhance the phosphorylation of STAT3, thus activating primary hepatic stellate cells and then aggravating hepatic fibrosis (Xiang et al., 2018). The binding of IL-6 to its receptor leads to the phosphorylation of intracellular signal molecule STAT3. Phospho-STAT3 dimerizes and then transfers to the nucleus, where it interacts with the characteristic IL-6 response element in the ferritin promoter (Fleming et al., 2011). Therefore, IL-6 might be the initiating factor of liver injury in our CUMS rat model, and then induced vacuolization of hepatocytes and increased COL I deposition by activating JAK2/STAT3 pathway. Indeed, with a check of phosphorylation of JAK2 and STAT3, we found that CUMS provoke a robust activation of JAK2 and STAT3 along with increased hepatic IL-6 levels, which were completely reversed by SX-1 treatment.

In our further study in AML12 cells, we applied CoCl_2_ to simulate cell hypoxia model, which can increase the expression of IL-6 in hepatocytes (Andrews and Arredondo, 2012). We chose the hypoxia model because we observed that CUMS induced the high expression of HIF-2α in animals, which is also consistent with the traditional Chinese medicine theory and the medicinal mechanism of SX-1. CUMS is a long-term process, which will cause liver qi stagnation (Wang et al., 2017). The main function of SX-1 is to soothe the liver and nourish blood. Sufficient liver blood can ensure sufficient oxygen to maintain the physiological function of the liver. It was also detected that the high expression of HIF-2α was inhibited in rat liver tissue, but there was no significant difference in other related proteins. Therefore, we used CoCl_2_ *in vitro* to simulate the environment *in vivo* and verify the mechanism in the environment of cell hypoxia. Consistently, in the CoCl_2_ induced hypoxia model of AML12 cells, we observed that SX-1 down regulated the protein expression level of IL-6 induced by CoCl_2_, and its down-regulation effect of p-STAT3 was not significantly different from that of stattic (Figure 7). This result further increased the possibility of SX-1 improving hepatocyte injury by blocking IL-6/JAK2/STAT3 signaling pathway.

The present study showed that SX-1 protected CUMS-induced depression-like and hepatic disorders. However, a conclusion of that SX-1 protection of depression-like behavior by inhibiting liver injury could not be drawn. In the theory of traditional Chinese medicine, catharsis of liver Qi is helpful to inhibit the occurrence and development of depression. Indeed, a close correlation ship has been shown between depression and hepatic disorders, such as non-alcoholic fatty liver disease and liver transplantation (Herzer et al., 2020; Funuyet-Salas et al., 2021). To verify the SX-1 protection of CUMS-induced depression-like behavior via inhibition of hepatic IL-6/JAK2/STAT3 signaling, we will repeat the present study in a mouse model of liver-specific knockout of IL-6 in the future.

## Conclusion

**FIGURE 8:**
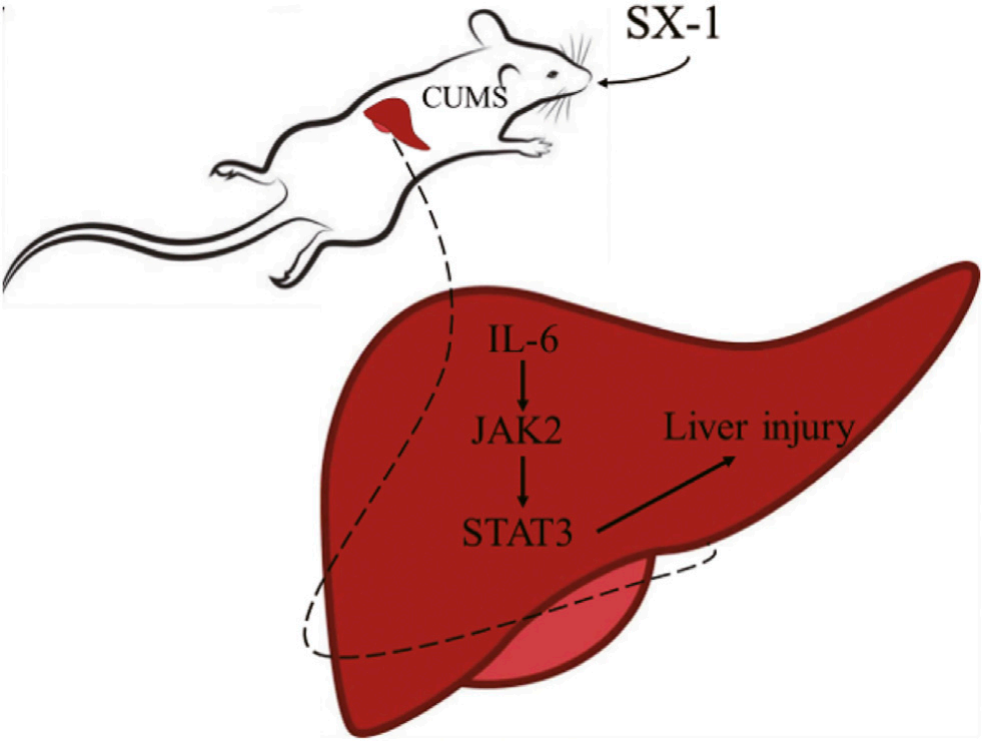
The mechanism of SX-1 on amelioration of CUMS-induced liver injury via inhibition of IL-6/JAK2/STAT3 Signaling.

CUMS induced hepatic injuries in addition to depression-like emotional disorders. SX-1decoction, a liver nourishing TCM formula, significantly ameliorated CUMS-induced hepatic injuries in addition to improving the depression-like behaviors. SX-1 inhibited the activation of hepatic IL-6/JAK2/STAT3 signaling, which might be the molecular mechanism mediating the SX-1 protection of CUMS-induced hepatic injuries (Figure 8). Thus SX-1 might be a promising strategy in treating CUMS- induced emotional disorders as well as hepatic injuries.

## Data Availability

The original contributions presented in the study are included in the article/Supplementary Material, further inquiries can be directed to the corresponding authors.

## References

[B1] Andrews M. Arredondo M. (2012). Hepatic and Adipocyte Cells Respond Differentially to Iron Overload, Hypoxic and Inflammatory challenge. Biometals 25 (4), 749–759. 10.1007/s10534-012-9543-9 22476617

[B2] Antoniuk S. Bijata M. Ponimaskin E. Wlodarczyk J. (2019). Chronic Unpredictable Mild Stress for Modeling Depression in Rodents: Meta-Analysis of Model Reliability. Neurosci. Biobehav. Rev. 99, 101–116. 10.1016/j.neubiorev.2018.12.002 30529362

[B3] Bartoszewski R. Moszyńska A. Serocki M. Cabaj A. Polten A. Ochocka R. (2019). Primary Endothelial Cell-specific Regulation of Hypoxia-Inducible Factor (HIF)-1 and HIF-2 and Their Target Gene Expression Profiles during Hypoxia. FASEB J. 33 (7), 7929–7941. 10.1096/fj.201802650RR 30917010 PMC6593883

[B4] Baumann H. Gauldie J. (1994). The Acute Phase Response. Immunol. Today 15 (2), 74–80. 10.1016/0167-5699(94)90137-6 7512342

[B5] Chen Y. L. Lee C. Y. Huang K. H. Kuan Y. H. Chen M. (2015). Prescription Patterns of Chinese Herbal Products for Patients with Sleep Disorder and Major Depressive Disorder in Taiwan. J. Ethnopharmacol 171, 307–316. 10.1016/j.jep.2015.05.045 26068429

[B6] Chen C. Yin Q. Tian J. Gao X. Qin X. Du G. (2020). Studies on the Potential Link between Antidepressant Effect of Xiaoyao San and its Pharmacological Activity of Hepatoprotection Based on Multi-Platform Metabolomics. J. Ethnopharmacol. 249, 112432. 10.1016/j.jep.2019.112432 31790818

[B7] Choi I. Kang H. S. Yang Y. Pyun K. H. (1994). IL-6 Induces Hepatic Inflammation and Collagen Synthesis *In Vivo* . Clin. Exp. Immunol. 95 (3), 530–535. 10.1111/j.1365-2249.1994.tb07031.x 8137551 PMC1535082

[B8] Chrousos G. P. George P. (2009). Stress and Disorders of the Stress System. Nat. Rev. Endocrinol. 5 (7), 374–381. 10.1038/nrendo.2009.106 19488073

[B9] Dang W. Z. Li H. Jiang B. Nandakumar K. S. Liu K. F. Liu L. X. (2019). Therapeutic Effects of Artesunate on Lupus-Prone MRL/lpr Mice Are Dependent on T Follicular Helper Cell Differentiation and Activation of JAK2-STAT3 Signaling Pathway. Phytomedicine 62, 152965. 10.1016/j.phymed.2019.152965 31129432

[B10] Demirdaş A. Nazıroğlu M. Ünal G. Ö. (2016). Agomelatine Reduces Brain, Kidney and Liver Oxidative Stress but Increases Plasma Cytokine Production in the Rats with Chronic Mild Stress-Induced Depression. Metab. Brain Dis. 31 (6), 1445–1453. 10.1007/s11011-016-9874-2 27438049

[B11] Duda W. Curzytek K. Kubera M. Iciek M. Kowalczyk-Pachel D. Bilska-Wilkosz A. (2016). The Effect of Chronic Mild Stress and Imipramine on the Markers of Oxidative Stress and Antioxidant System in Rat Liver. Neurotox Res. 30 (2), 173–184. 10.1007/s12640-016-9614-8 26961706 PMC4947122

[B12] Fleming R. E. Feng Q. Britton R. S. (2011). Knockout Mouse Models of Iron Homeostasis. Annu. Rev. Nutr. 31, 117–137. 10.1146/annurev-nutr-072610-145117 21548776

[B13] Funuyet-Salas J. Martín-Rodríguez A. Pérez-San-Gregorio M. Á. Romero-Gómez M. (2021). Influence of Psychological Biomarkers on Therapeutic Adherence by Patients with Non-alcoholic Fatty Liver Disease: A Moderated Mediation Model. J. Clin. Med. 10 (10), 2208. 10.3390/jcm10102208 34065216 PMC8161151

[B14] Gabay C. Kushner I. (1999). Acute-phase Proteins and Other Systemic Responses to Inflammation. N. Engl. J. Med. 340 (6), 448–454. 10.1056/NEJM199902113400607 9971870

[B15] Gao Y. Zhou Z. Ren T. Kim S. J. He Y. Seo W. (2019). Alcohol Inhibits T-Cell Glucose Metabolism and Hepatitis in ALDH2-Deficient Mice and Humans: Roles of Acetaldehyde and Glucocorticoids. Gut 68 (7), 1311–1322. 10.1136/gutjnl-2018-316221 30121625 PMC6582747

[B16] Gu F. M. Li Q. L. Gao Q. Jiang J. H. Zhu K. Huang X. Y. (2011). IL-17 Induces AKT-dependent IL-6/JAK2/STAT3 Activation and Tumor Progression in Hepatocellular Carcinoma. Mol. Cancer 10, 150. 10.1186/1476-4598-10-150 22171994 PMC3310750

[B17] Gu Y. Liu W. Liu G. Li X. Lu P. (2021). Assessing the Protective Effects of Cryptotanshinone on CoCl_2_-induced Hypoxia in RPE Cells. Mol. Med. Rep. 24 (4), 739. 10.3892/mmr.2021.12379 34435647 PMC8404095

[B18] Guo L.-T. Wang S.-Q. Su J. Xu L.-X. Ji Z.-Y. Zhang R.-Y. (2019). Baicalin Ameliorates Neuroinflammation-Induced Depressive-like Behavior through Inhibition of Toll-like Receptor 4 Expression via the PI3K/AKT/FoxO1 Pathway. J. Neuroinflammation 16 (1), 95. 10.1186/s12974-019-1474-8 31068207 PMC6507025

[B19] Herzer K. Sterneck M. Welker M. W. Nadalin S. Kirchner G. Braun F. (2020). Current Challenges in the Post-Transplant Care of Liver Transplant Recipients in Germany. J. Clin. Med. 9 (11), 3570. 10.3390/jcm9113570 PMC7694452 33167567

[B20] Ilamathi M. Prabu P. C. Ayyappa K. A. Sivaramakrishnan V. (2016). Artesunate Obliterates Experimental Hepatocellular Carcinoma in Rats through Suppression of IL-6-JAK-STAT Signalling. Biomed. Pharmacother. 82, 72–79. 10.1016/j.biopha.2016.04.061 27470341

[B21] Jia H. M. Yu M. Ma L. Y. Zhang H. W. Zou Z. M. (2017). Chaihu-Shu-Gan-San Regulates Phospholipids and Bile Acid Metabolism against Hepatic Injury Induced by Chronic Unpredictable Stress in Rat. J. Chromatogr. B Analyt Technol. Biomed. Life Sci. 1064, 14–21. 10.1016/j.jchromb.2017.08.003 28886478

[B22] Johnson D. E. O'Keefe R. A. Grandis J. R. (2018). Targeting the IL-6/JAK/STAT3 Signalling axis in Cancer. Nat. Rev. Clin. Oncol. 15 (4), 234–248. 10.1038/nrclinonc.2018.8 29405201 PMC5858971

[B23] Kortam M. A. Ali B. M. Fathy N. (2021). The Deleterious Effect of Stress-Induced Depression on Rat Liver: Protective Role of Resveratrol and Dimethyl Fumarate via Inhibiting the MAPK/ERK/JNK Pathway. J. Biochem. Mol. Toxicol. 35 (1), e22627. 10.1002/jbt.22627 32905656

[B24] Li Q. Yang H. Wang W. Li N. Zou X. Li Y. (2020). Brassica Rapa Polysaccharides Ameliorate CCl4 -Induced Acute Liver Injury in Mice through Inhibiting Inflammatory Apoptotic Response and Oxidative Stress. Chem. Biodivers 17 (1), e1900534. 10.1002/cbdv.201900534 31730730

[B25] Liu X. Lv M. Wang Y. Zhao D. Zhao S. Li S. (2020). Deciphering the Compatibility Rules of Traditional Chinese Medicine Prescriptions Based on NMR Metabolomics: A Case Study of Xiaoyaosan. J. Ethnopharmacol. 254, 112726. 10.1016/j.jep.2020.112726 32135241

[B26] Miller E. S. Apple C. G. Kannan K. B. Funk Z. M. Plazas J. M. Efron P. A. (2019). Chronic Stress Induces Persistent Low-Grade Inflammation. Am. J. Surg. 218 (4), 677–683. 10.1016/j.amjsurg.2019.07.006 31378316 PMC6768696

[B27] Nair A. Morsy M. A. Jacob S. (2018). Dose Translation between Laboratory Animals and Human in Preclinical and Clinical Phases of Drug Development. Drug Dev. Res. 79 (8), 373–382. 10.1002/ddr.21461 30343496

[B28] Papanicolaou D. A. Tsigos C. Oldfield E. H. Chrousos G. P. (1996). Acute Glucocorticoid Deficiency Is Associated with Plasma Elevations of Interleukin-6: Does the Latter Participate in the Symptomatology of the Steroid Withdrawal Syndrome and Adrenal Insufficiency? J. Clin. Endocrinol. Metab. 81 (6), 2303–2306. 10.1210/jcem.81.6.8964868 8964868

[B29] Papp M. Willner P. Muscat R. (1991). An Animal Model of Anhedonia: Attenuation of Sucrose Consumption and Place Preference Conditioning by Chronic Unpredictable Mild Stress. Psychopharmacology (Berl) 104 (2), 255–259. 10.1007/BF02244188 1876670

[B30] Qu A. Taylor M. Xue X. Matsubara T. Metzger D. Chambon P. (2011). Hypoxia-inducible Transcription Factor 2α Promotes Steatohepatitis through Augmenting Lipid Accumulation, Inflammation, and Fibrosis. Hepatology 54 (2), 472–483. 10.1002/hep.24400 21538443 PMC3145012

[B31] Quinn A. M. Williams A. R. Sivilli T. I. Raison C. L. Pace T. W. W. (2018). The Plasma Interleukin-6 Response to Acute Psychosocial Stress in Humans Is Detected by a Magnetic Multiplex Assay: Comparison to High-Sensitivity ELISA. Stress 21 (4), 376–381. 10.1080/10253890.2018.1446518 29529950 PMC6662910

[B32] Schmidt-Arras D. Rose-John S. (2016). IL-6 Pathway in the Liver: From Physiopathology to Therapy. J. Hepatol. 64 (6), 1403–1415. 10.1016/j.jhep.2016.02.004 26867490

[B33] Sturman O. Germain P. L. Bohacek J. (2018). Exploratory Rearing: a Context- and Stress-Sensitive Behavior Recorded in the Open-Field Test. Stress 21 (5), 443–452. 10.1080/10253890.2018.1438405 29451062

[B34] Tang Q. Ran H. (2018). MicroRNA-219-5p Inhibits Wound Healing by Targeting TMEM98 in Keratinocytes under Normoxia and Hypoxia Condition. Eur. Rev. Med. Pharmacol. Sci. 22 (19), 6205–6211. 10.26355/eurrev_201810_16026 30338788

[B35] van Gool J. van Vugt H. Helle M. Aarden L. A. (1990). The Relation Among Stress, Adrenalin, Interleukin 6 and Acute Phase Proteins in the Rat. Clin. Immunol. Immunopathol. 57 (2), 200–210. 10.1016/0090-1229(90)90034-n 1698583

[B36] Wang H. Zhang Y. Li H. Zeng W. Qiao M. (2017). Shuyu Capsules Relieve Liver-Qi Depression by Regulating ERK-CREB-BDNF Signal Pathway in central Nervous System of Rat. Exp. Ther. Med. 14 (5), 4831–4838. 10.3892/etm.2017.5125 29201187 PMC5704346

[B37] Xiang D. M. Sun W. Ning B. F. Zhou T. F. Li X. F. Zhong W. (2018). The HLF/IL-6/STAT3 Feedforward Circuit Drives Hepatic Stellate Cell Activation to Promote Liver Fibrosis. Gut 67 (9), 1704–1715. 10.1136/gutjnl-2016-313392 28754776

[B38] Yi P. L. TsaiChen C. H. Chen Y. C. Chang F. C. (2007). Gamma-aminobutyric Acid (GABA) Receptor Mediates Suanzaorentang, a Traditional Chinese Herb Remedy, -induced Sleep Alteration. J. Biomed. Sci. 14 (2), 285–297. 10.1007/s11373-006-9137-z 17151826

[B39] Youssef N. A. Abdelmalek M. F. Binks M. Guy C. D. Omenetti A. Smith A. D. (2013). Associations of Depression, Anxiety and Antidepressants with Histological Severity of Nonalcoholic Fatty Liver Disease. Liver Int. 33 (7), 1062–1070. 10.1111/liv.12165 23560860

[B40] Zhou Y. Wu R. Cai F. F. Zhou W. J. Lu Y. Y. Zhang H. (2021). Xiaoyaosan Decoction Alleviated Rat Liver Fibrosis via the TGFβ/Smad and Akt/FoxO3 Signaling Pathways Based on Network Pharmacology Analysis. J. Ethnopharmacol. 264, 113021. 10.1016/j.jep.2020.113021 32479885

[B41] Zhu H. P. Gao Z. L. Tan D. M. Zhong Y. D. (2007). Effect of Suanzaoren Decoction on Acute Hepatic Failure in Mice. Zhongguo Zhong Yao Za Zhi 32 (8), 718–721. 17608229

