# Supplementary material for: Shuxie-1 Decoction Alleviated CUMS -Induced Liver Injury via IL-6/JAK2/STAT3 Signaling
Source: Front Pharmacol. 2022 Apr 6;13:848355. doi: 10.3389/fphar.2022.848355 (PMC9019685; doi:10.3389/fphar.2022.848355)
Supplement: Supplementary file 1 [file Presentation1.PPTX]

## Slide 1
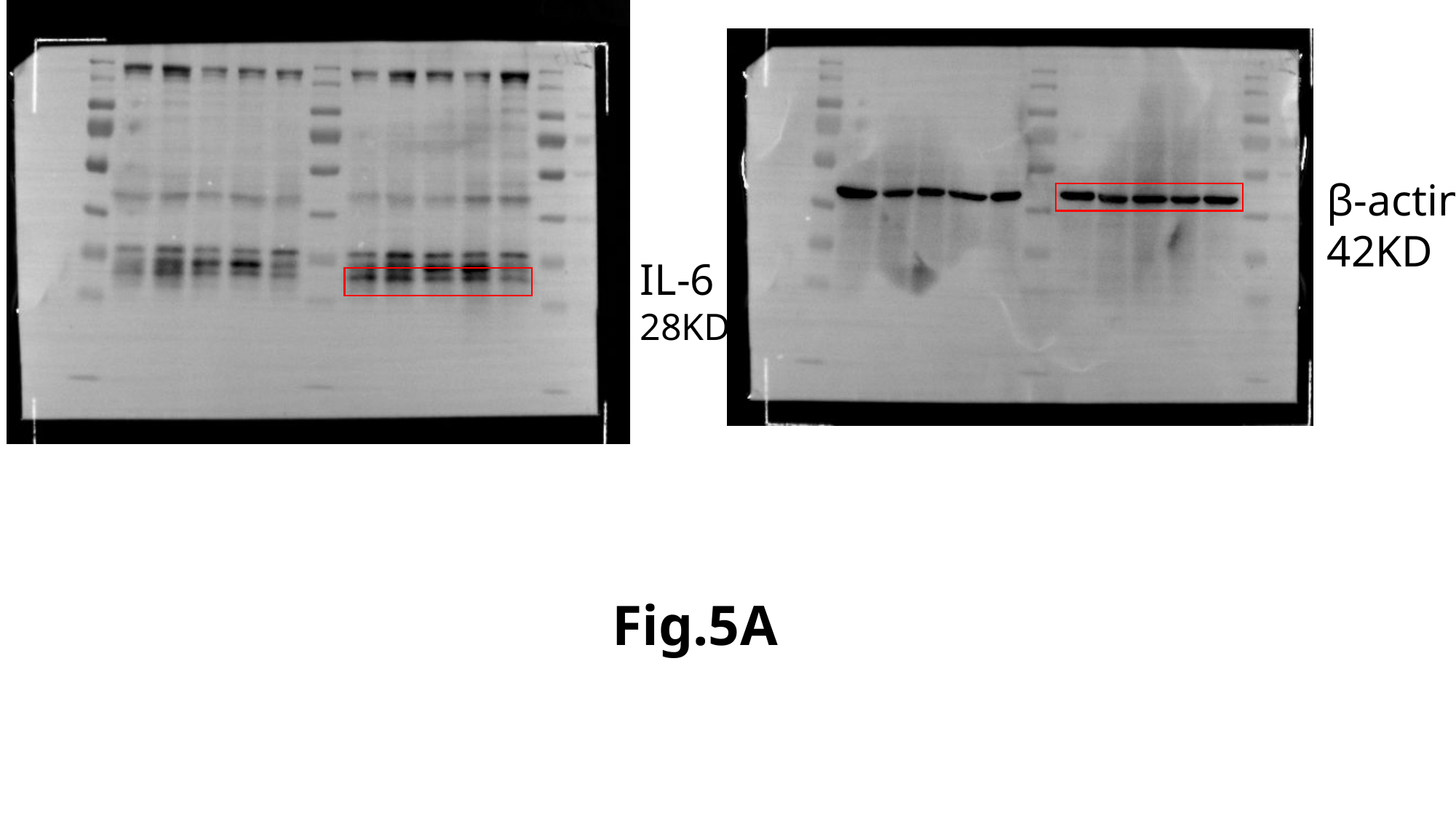

β-actin
42KD
IL-6
28KD
Fig.5A

## Slide 2
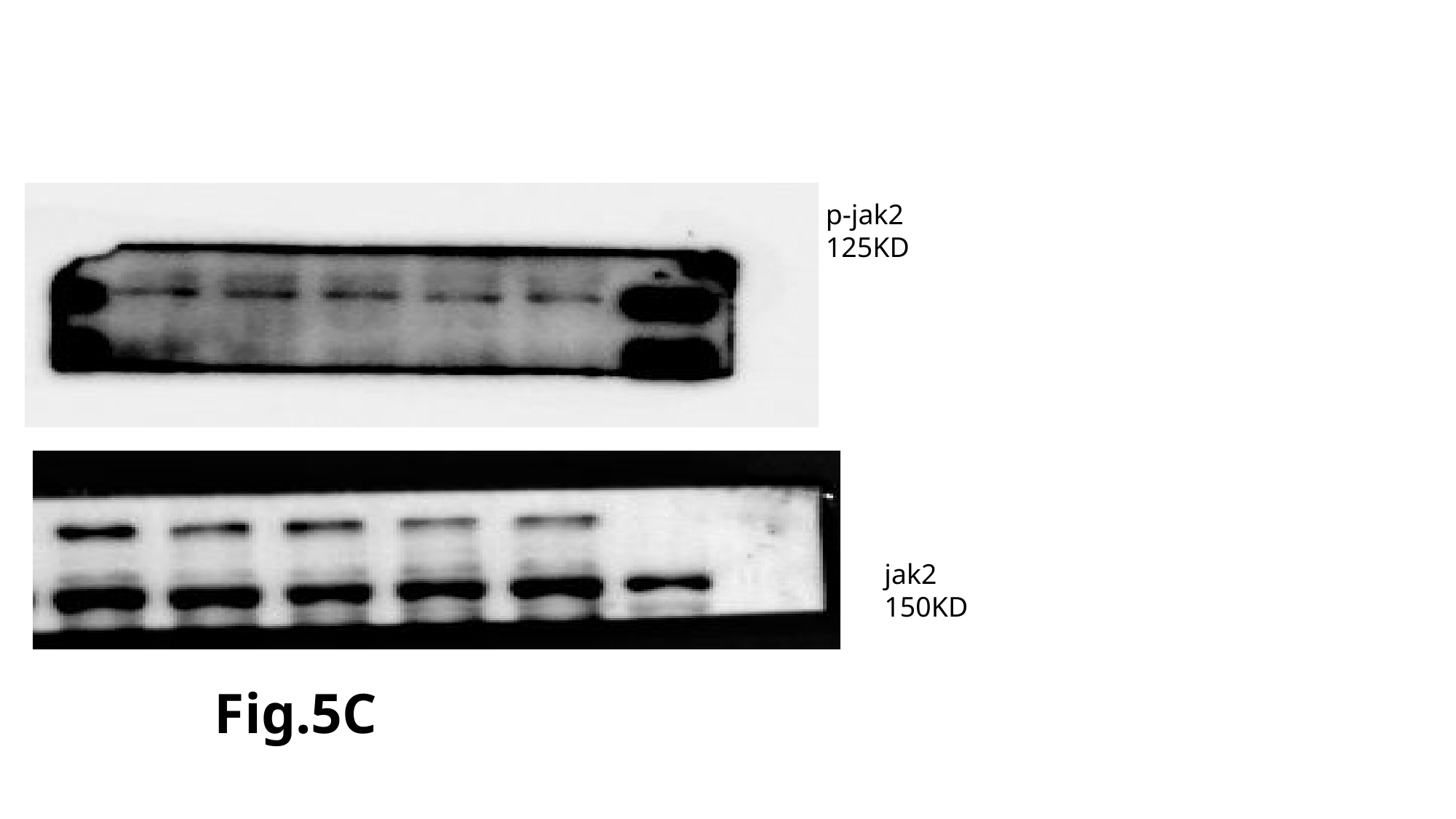

p-jak2
125KD
jak2
150KD
Fig.5C

## Slide 3
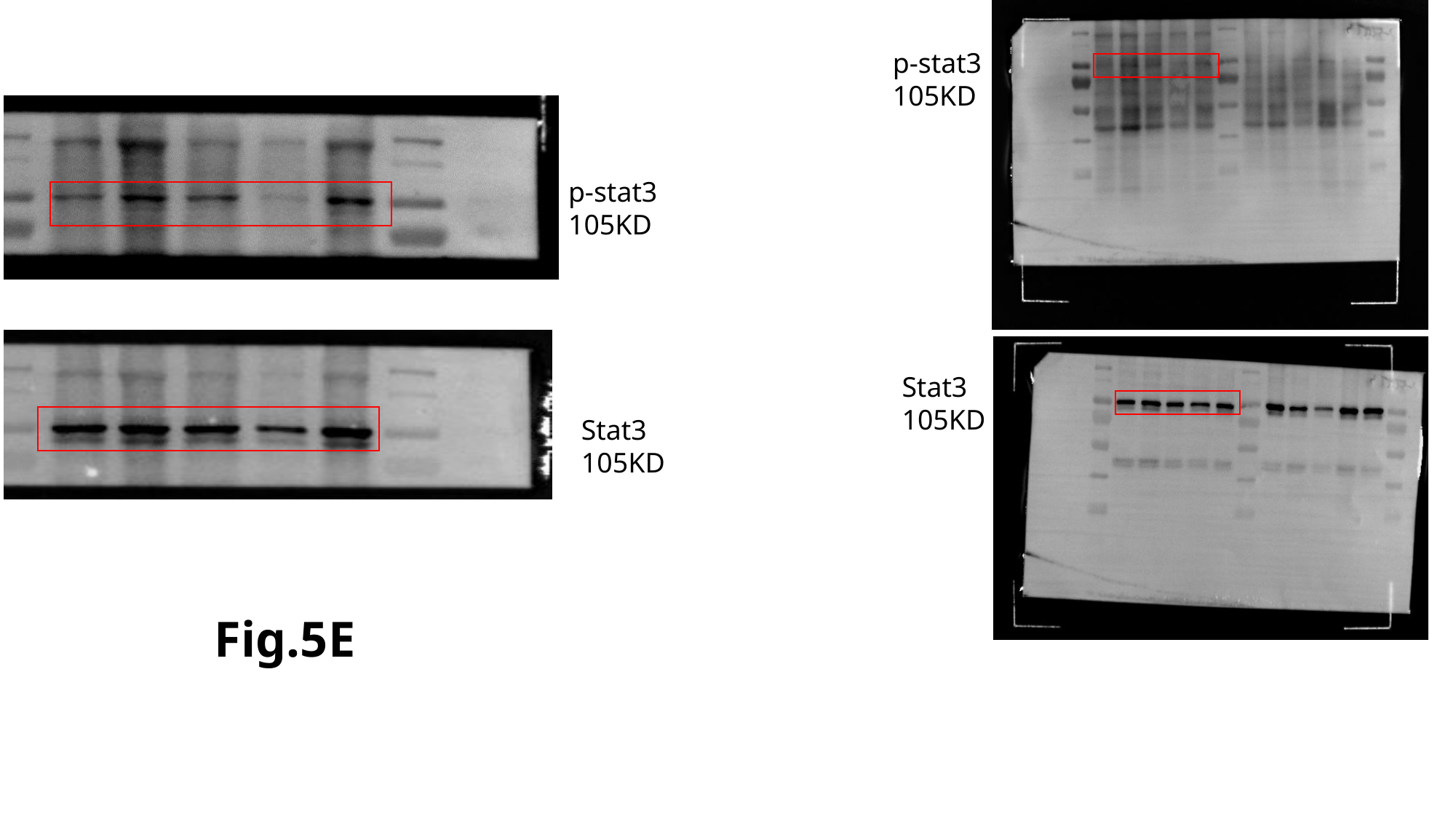

p-stat3
105KD
p-stat3
105KD
Stat3
105KD
Stat3
105KD
Fig.5E

## Slide 4
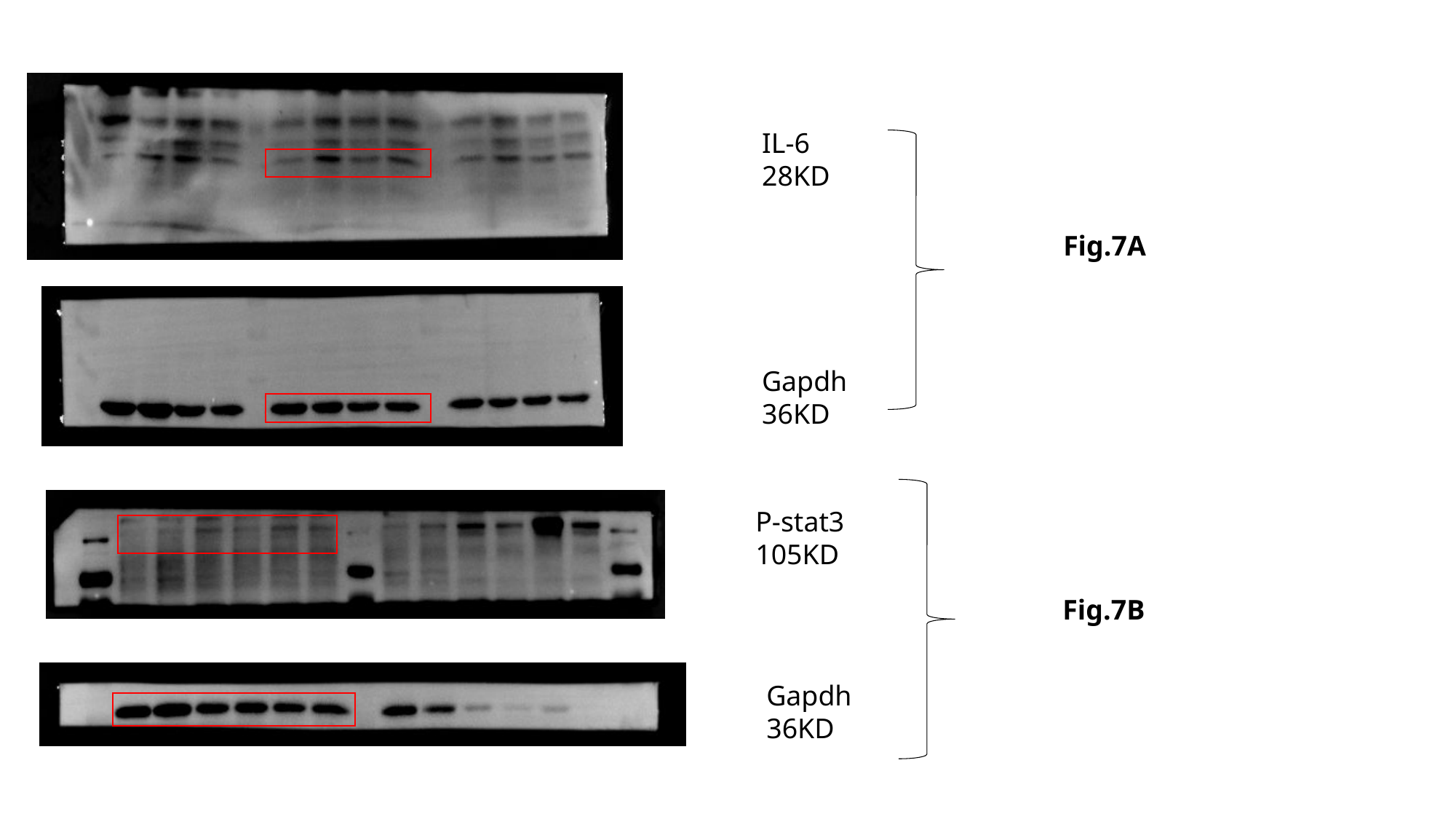

IL-6
28KD
Fig.7A
Gapdh
36KD
P-stat3
105KD
Fig.7B
Gapdh
36KD

## Slide 5
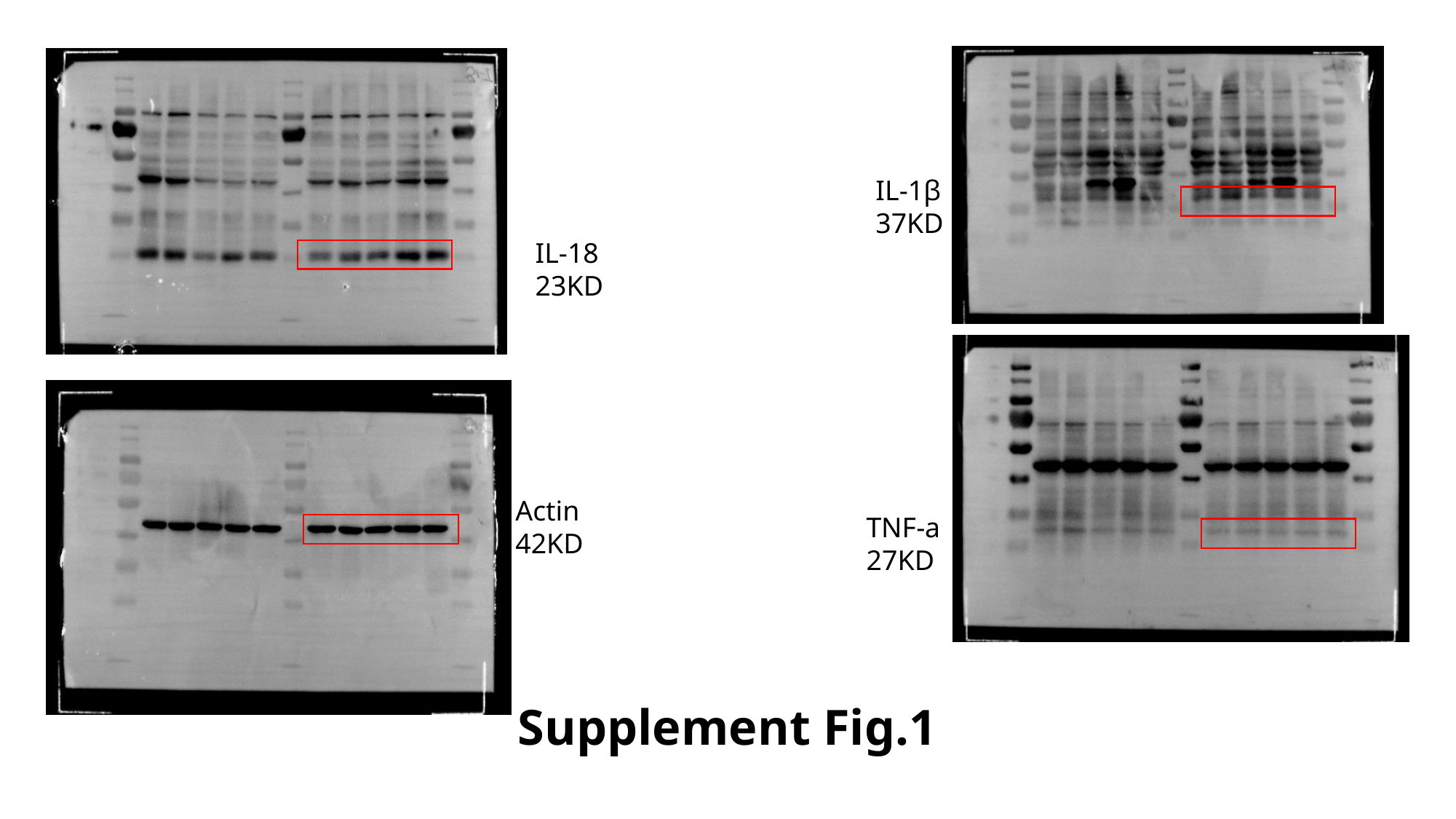

IL-1β
37KD
IL-18
23KD
Actin
42KD
TNF-a
27KD
Supplement Fig.1

## Slide 6
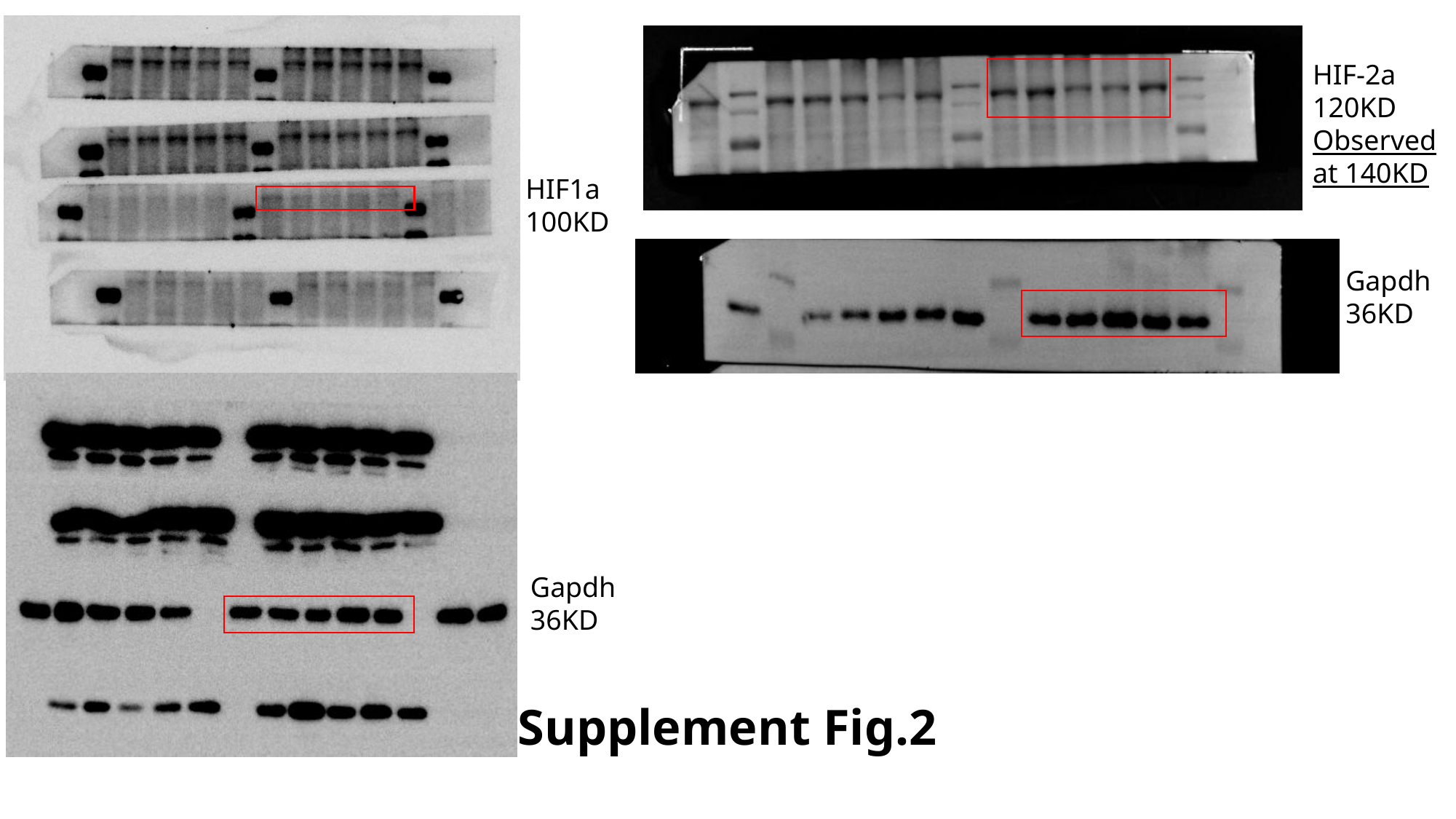

HIF-2a
120KD
Observed at 140KD
HIF1a
100KD
Gapdh
36KD
Gapdh
36KD
Supplement Fig.2

## Slide 7
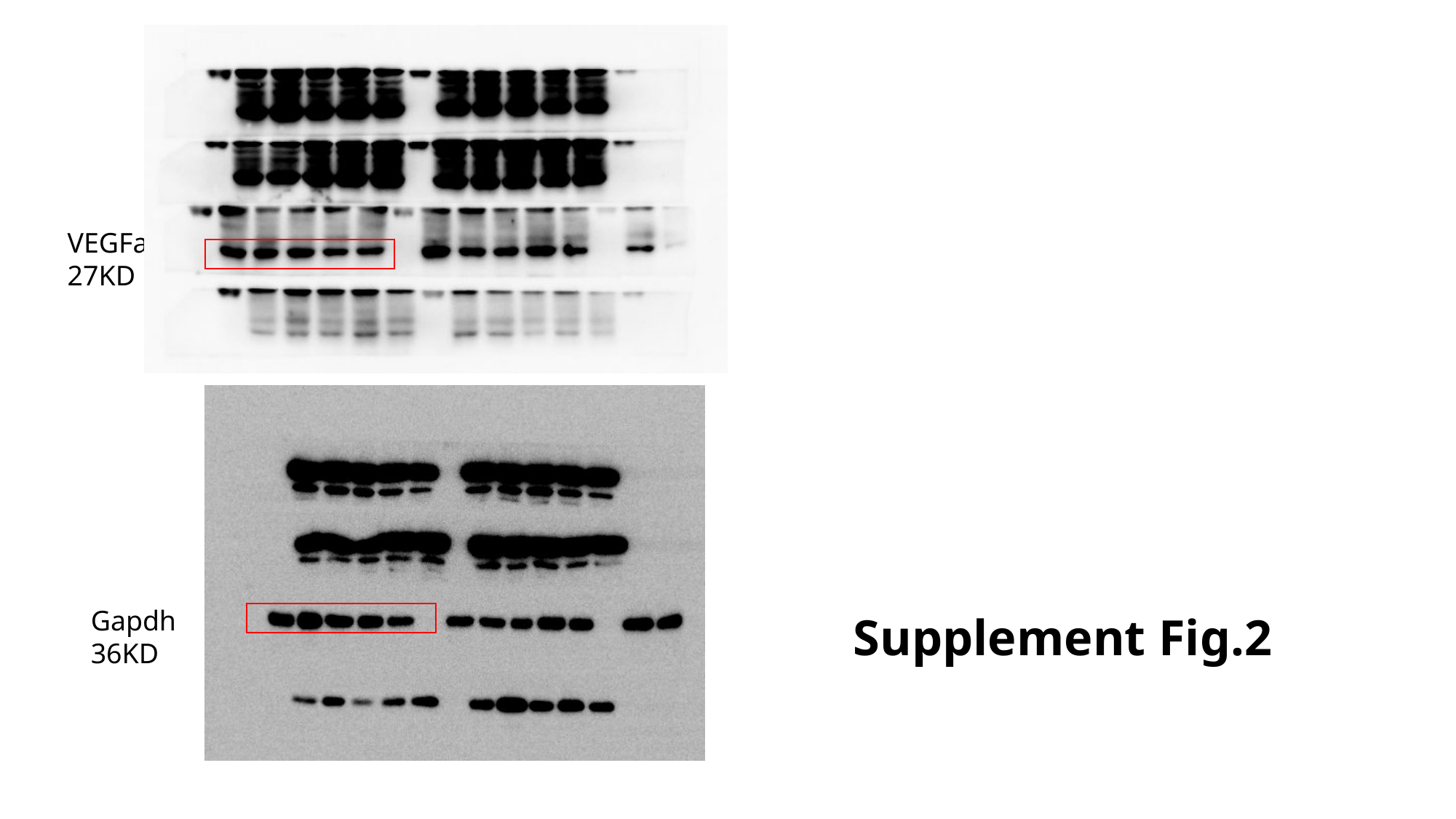

VEGFa
27KD
Gapdh
36KD
Supplement Fig.2

## Slide 8
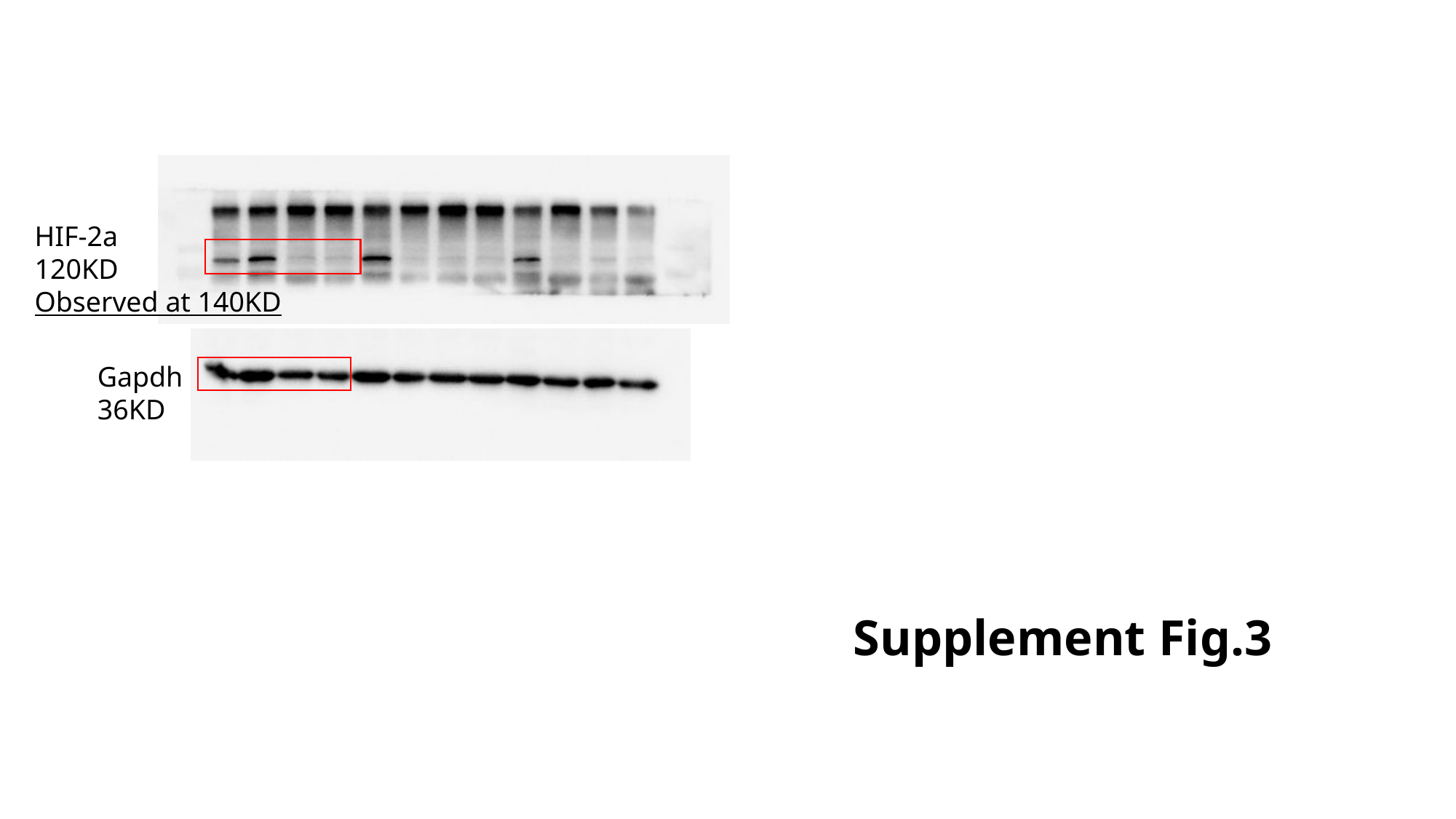

HIF-2a
120KD
Observed at 140KD
Gapdh
36KD
Supplement Fig.3
