# Supplementary material for: Shuxie-1 Decoction Alleviated CUMS -Induced Liver Injury via IL-6/JAK2/STAT3 Signaling
Source: Front Pharmacol. 2022 Apr 6;13:848355. doi: 10.3389/fphar.2022.848355 (PMC9019685; doi:10.3389/fphar.2022.848355)
Supplement: Supplementary file 2 [file DataSheet1.DOCX]

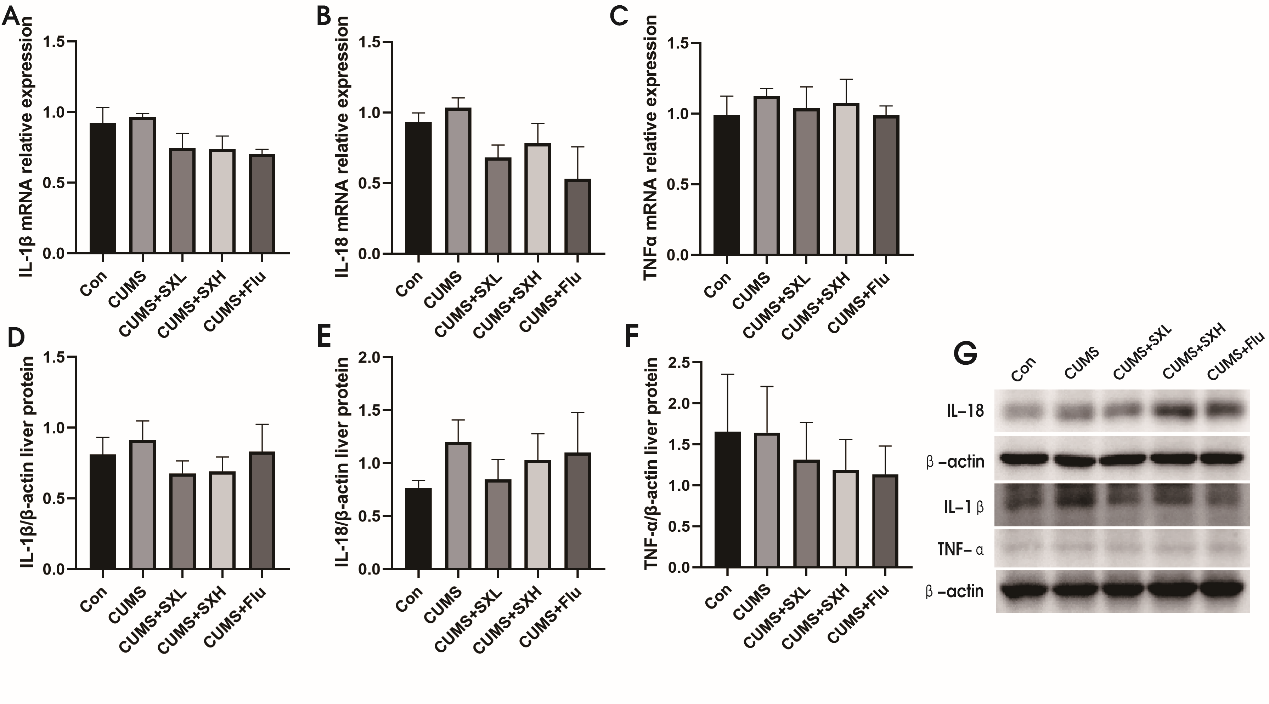


Supplement Figure 1 IL-1β, IL-18, TNF-α mRNA relative expression and protein expression in liver tissue. mRNA and protein level of IL-1β, IL-18, TNF-α were detected by qPCR and western blot respectively (A-G).


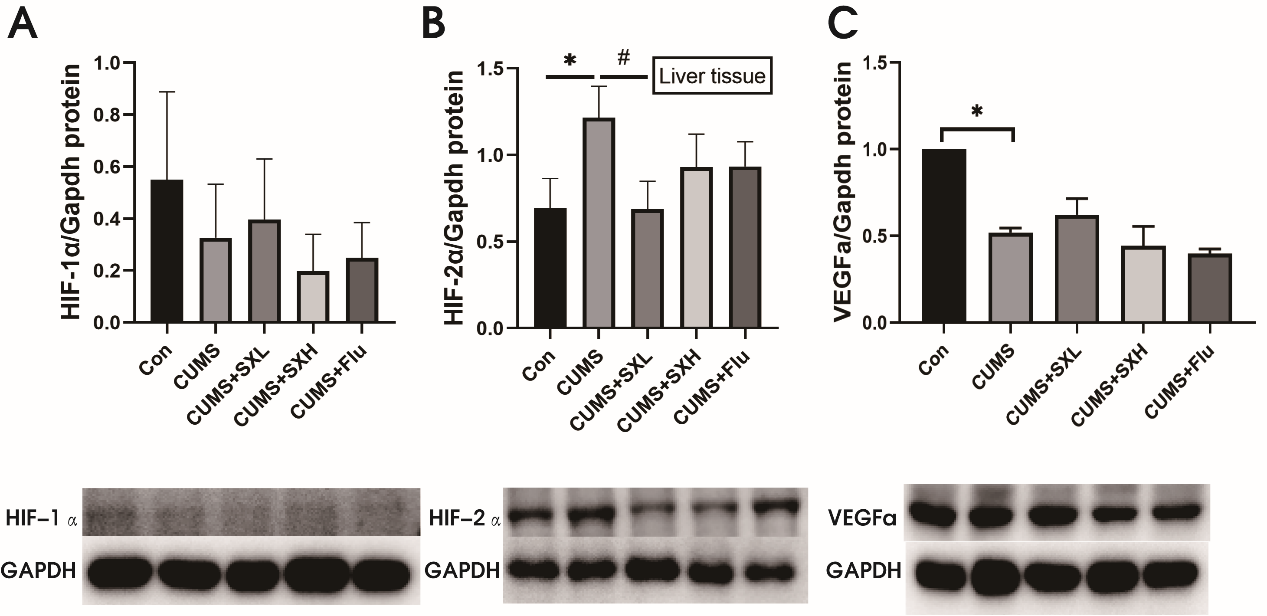


Supplement Figure 2 HIF-1α, HIF-2α and VEGFa protein expression in liver tissue. HIF-1α, HIF-2α and VEGFa in liver tissue was detected by western blot. *: compared to control group (Con); #: compared to CUMS group.


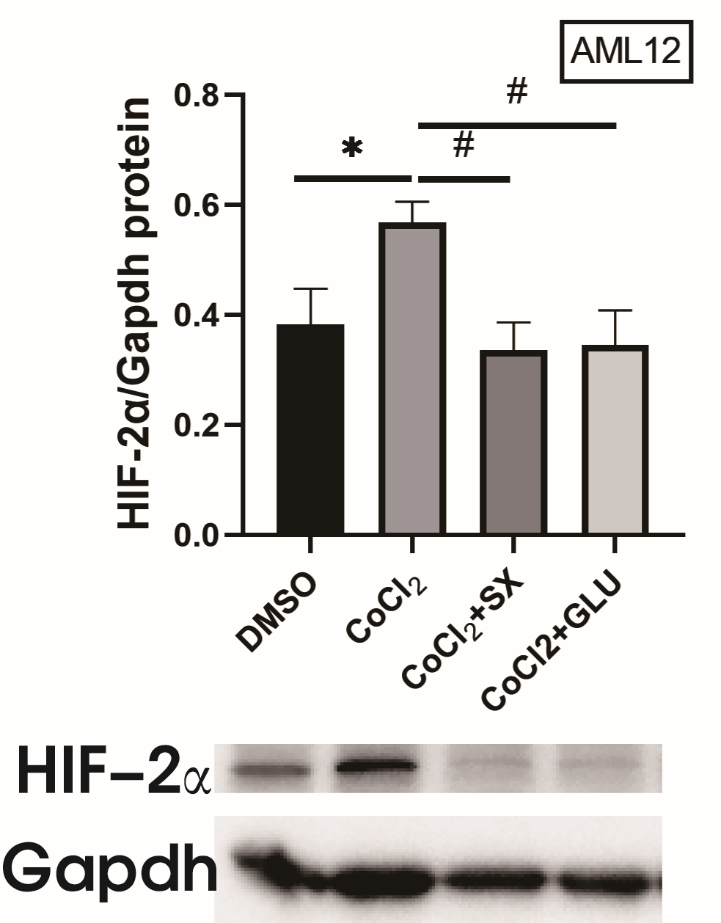


Supplement Figure 3 HIF-2α protein expression in AML12 cell. HIF-2α in AML12 cell was detected by western blot. *: compared to DMSO group; #: compared to CoCl_2_ group.
